# Bio-Based Polymer Electrolytes for Electrochemical Devices: Insight into the Ionic Conductivity Performance

**DOI:** 10.3390/ma13040838

**Published:** 2020-02-12

**Authors:** Marwah Rayung, Min Min Aung, Shah Christirani Azhar, Luqman Chuah Abdullah, Mohd Sukor Su’ait, Azizan Ahmad, Siti Nurul Ain Md Jamil

**Affiliations:** 1 Institute of Tropical Forestry and Forest Products, Universiti Putra Malaysia, Serdang 43400, Malaysia; marwahrayung@yahoo.com; 2 Unit Chemistry, Center of Foundation Studies and Agricultural Science, Universiti Putra Malaysia, Serdang 43400, Malaysia; christirani@upm.edu.my (S.C.A.); ctnurulain@upm.edu.my (S.N.A.M.J.); 3 Department of Chemical and Environmental Engineering, Faculty of Engineering, Universiti Putra Malaysia, Serdang 43400, Malaysia; chuah@upm.edu.my; 4 Solar Energy Research Institute (SERI), Universiti Kebangsaan Malaysia, Bangi 43600, Malaysia; mohdsukor@ukm.edu.my (M.S.S.); azizan@ukm.edu.my (A.A.); 5 School of Chemical Sciences and Food Technology, Universiti Kebangsaan Malaysia, Bangi 43600, Malaysia

**Keywords:** bio-based polymer, polymer electrolyte, ionic conductivity, electrochemical devices

## Abstract

With the continuing efforts to explore alternatives to petrochemical-based polymers and the escalating demand to minimize environmental impact, bio-based polymers have gained a massive amount of attention over the last few decades. The potential uses of these bio-based polymers are varied, from household goods to high end and advanced applications. To some extent, they can solve the depletion and sustainability issues of conventional polymers. As such, this article reviews the trends and developments of bio-based polymers for the preparation of polymer electrolytes that are intended for use in electrochemical device applications. A range of bio-based polymers are presented by focusing on the source, the general method of preparation, and the properties of the polymer electrolyte system, specifically with reference to the ionic conductivity. Some major applications of bio-based polymer electrolytes are discussed. This review examines the past studies and future prospects of these materials in the polymer electrolyte field.

## 1. Introduction to Bio-Based Polymers

Bio-based polymers are growing in importance over the past few decades due to their potential as replacements or alternatives to conventional polymers. They are the key feature to solve many international issues such as global warming, price fluctuations, the shortage of petroleum resources, pollution, and other economic and ecological issues. In general, bio-based polymers refer to a type of polymer that is produced naturally by living organisms [1]. In other words, it is also called a natural polymer. This type of polymer has the following characteristics: Using natural raw materials as base materials, non-toxic, biodegradable, and sustainable.

**Figure 1:**
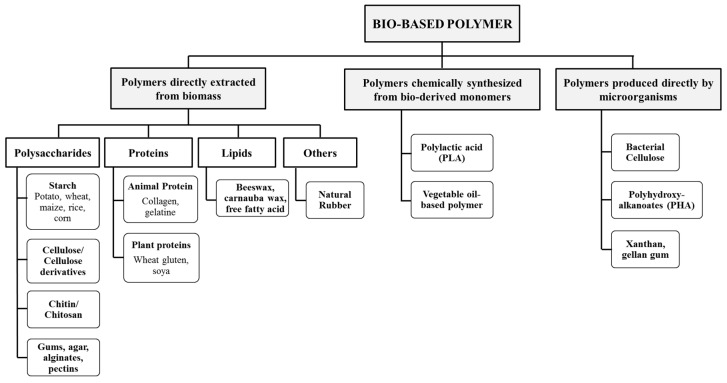
Classification of bio-based polymer (adapted from Malhotra et al. [2]).

Bio-based polymers can be classified into three main categories based on their synthesis and origin of source. The first refers to polymers directly extracted from biomass, such as starch, cellulose, chitosan, and alginates. They are the most abundant and a major resource of bio-based polymers. The second category concerns polymers synthesized from bio-derived monomers, and the third includes polymers synthesized by microorganisms/bacteria. Figure 1 illustrates the classification of bio-based polymers with examples [2].

The development and innovation of said materials are important, as they hold great potential for research studies and industrial applications. Table 1 presents the most common bio-based polymers, their sources, and related industrial applications. Many studies and development strategies have been devised to discover and to optimize the potential uses of this type of polymer for commercial applications, including the food packaging industry, agricultural purposes, cosmetics, the medical industry, and the pharmaceutical industry. This review offers an overview of bio-based polymers and their applications within the polymer electrolyte field.

## 2. Insights to the Polymer Electrolyte

**Figure 2:**
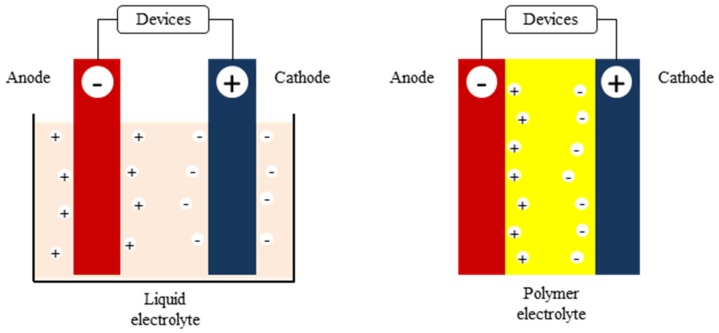
Diagram of a typical electrochemical cell.

An electrolyte is a significant element in developing electrochemical devices. In general, an electrolyte functions as a medium that allows the flow of ions between a cathode and an anode. An electrolyte also functions as an electronic insulator when the devices fail to work. It is essential to have an electrolyte with a sufficiently high value of ionic conductivity, preferably much higher than 10^−4^ S/cm [4]. The electrolyte can be a liquid, a gel, or in a solid form. Although the liquid electrolyte still dominates in many applications, it has several limitations such as flammability issues, leaking, reaction with the electrodes, and corrosion. A polymer electrolyte (PE) offers desirable properties to overcome the problems due to the all solid state condition. It is also inherently safer, as there is no flow and corrosion after damage, it has a wider electrochemical and thermal stability range, it is light weight, and has ease of application to electrochemical devices [5]. In fact, continuous efforts are being taken on polymer electrolytes as they have a great potential to be applied in a wide range of electrochemical devices. Figure 2 shows the use of a conventional electrolyte and polymer electrolyte in a typical electrochemical cell [6].

Polymer electrolytes (PEs) are a highly specialized multidisciplinary field that cuts across the disciplines of electrochemistry, polymer science, organic chemistry, and inorganic chemistry. In principle, a PE is made up of inorganic salt(s) dispersed in a polymer matrix forming a conducting solid system. The inorganic salt dissociates into ions and contributes to the conductivity. The first discovery of a polymer electrolyte was reported on poly(ethylene oxide) as the polymer host doped with alkali metal ions back in 1973 [7]. The results of the extensive characterization of electrolytes based on poly(ethylene oxide) (PEO), or hosts with similar chemical structures and a great variety of guest salt species, confirmed that many factors, including the choice of electrolyte components, preparative conditions, and thermal history, determine the electrochemical, thermal, and mechanical properties of the electrolyte system. The performance of polymer electrolytes is primarily evaluated based on their ionic conductivity and ion transport properties, which depend on many factors such as the mobility of the polymer chains, the dielectric constant of the polymer host, the degree of salt dissociation and its concentration, as well as the degree of ion aggregation [6].

**Figure 3:**
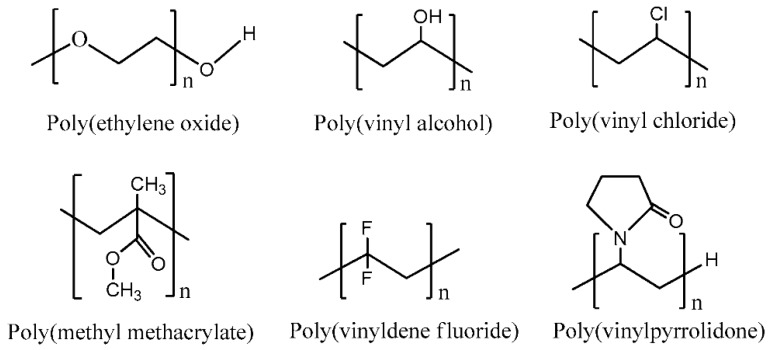
Chemical structure of some polar polymers used as a host polymer.

As for the construction of polymer electrolytes, several factors should be taken into consideration, including the choice of polymer host, the salts/acid dopants (the source of ions), the solvents, and other additives. The polymer host should possess certain characteristics such as good chemical, electrochemical, and photochemical stability, as well as good thermal and mechanical properties. Further, host polymers with a high concentration of polar groups (containing electron donors: O, NH, CN, F) are preferred. It is important to develop host polymers which have few crystalline phases and a relatively low glass transition temperature. In the amorphous state, greater ionic diffusivity may occur, as ions can move freely due to the low energy barrier. In addition, an amorphous polymer exhibits a flexible backbone that can increase local chain mobility. As a result, the segmental motion of a polymer can enhance the transportation property of the electrolyte [8]. Figure 3 shows the chemical structure of some polar polymers that are widely used as polymer hosts [9].

Another crucial aspect is the selection of salts. Salts provide the charge carriers for transportation that generate the conductivity [10]. The most commonly used salts are the salts of alkali metals, alkaline earth metals, and transition metals. The metal cations coordinate with the polar group from the polymer host. The segmental motion of the polymer chains creates free volume into which the ions will migrate and hence create the conductivity. The salts affect the ionic conductivity via several aspects, including complex formation, intramolecular cross-linking of the polymer chains, and the degree of salt dissociation. Apart from a polymer doped with metal salts, several studies of proton conducting electrolytes have been reported. Typically, a polymer is swollen with a solution of proton donors in a polar solvent containing redox sites [11]. As for the electrolyte solvent, it should first satisfy certain criteria. An ideal solvent should be able to dissolve salts to a sufficient concentration, with a high dielectric constant, low vapor pressure, low viscosity so that ion transport can occur, and to be inert to the electrodes.

More than two decades after the introduction of the polymer electrolyte concept, researchers have begun exploring alternatives to conventional polymer hosts derived from petroleum by utilizing bio-based materials as the polymer host. Even though most of the polymer electrolyte theories developed to date are based on synthetic materials, they hold true for bio-based polymers as well. Different types of bio-based polymers, thus, have been explored, such as chitosan, starch, carrageenan, gum, gelatin, natural rubber, and vegetable oil-based polymers. In addition, various electrolyte systems have been investigated with different types of salts, plasticizers, fillers, and ionic liquids. The priority remains the same, which is to produce a polymer electrolyte with a high conductivity, along with good thermal and mechanical properties. The types and characteristics of bio-based polymers, along with the outcomes, are discussed and summarized in the following section.

## 3. Bio-Based Polymers Used as Electrolytes

### 3.1. Polymers Extracted from Biomass

The first category of bio-based polymers is those directly extracted from biomass resources, including polysaccharides, proteins, lipids, and natural rubber. Based on the literature, polymers belonging to the polysaccharide group are the most studied in the polymer electrolyte field. They are easily accessible, widely available, and abundant. It is a fact that plants are the most important producers of polysaccharides. This section discusses the polysaccharides, proteins, and natural rubber-based polymer electrolytes.

#### 3.1.1. Starch

**Figure 4:**
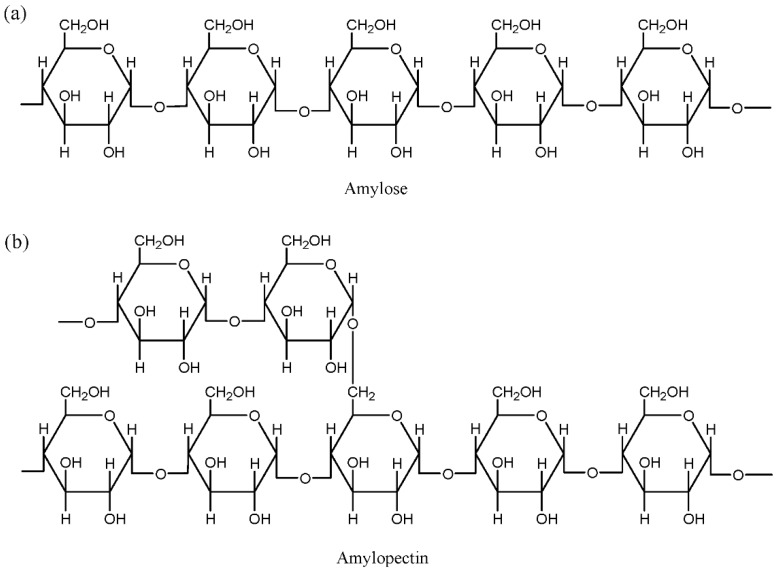
Representative units of starch (**a**) amylose and (**b**) amylopectin.

Extensive studies have probed starch on account of its rich variety, biodegradability, availability, and abundance in nature. Starch is the end-product of photosynthesis in plants. It is a natural carbohydrate-based polymer that is mainly harvested from corn, potato, wheat, tapioca, and rice [10]. The application of starch does not stop in major food products, but has been extended to various diversified areas. Starch is used in other applications as binders, adhesives, absorbents, and encapsulants [12]. Natural starch is a mixture of linear amylose or poly(α-1,4-d-glucopyranose) and α-1,6-branched amylopectin, wherein their components might varied depending on the plant sources [12,13]. Figure 4 illustrates the structures of amylose and amylopectin. Since these two compounds contain hydroxyl groups, the starch-based polymer emerges as a viable option to be used as a polymer host for electrolyte purposes.

Various types of starch have been investigated for polymer electrolyte studies, such as corn starch, cassava starch, arrowroot starch, sago starch, potato starch, rice starch, and tapioca starch. The starch has also been blended with other polymers such as PEO, chitosan, poly(styrene sulphonic acid), PVA, and methyl cellulose. The initial study concerning starch-based electrolyte was reported by Pawlicka et al. in 2002 for a corn starch/LiClO_4_/glycerol system [14]. Aside from glycerol, other types of plasticizers have been used, such as glucose, sorbitol, urea, formamide, glutaraldehyde, and ethylene carbonate. Additionally, various types of salts have been used as well. The effect of ionic liquid inclusion on the ionic conductivity of a polymer electrolyte has also been investigated. Inorganic fillers, such as silicon dioxide, barium titanate, and graphene oxide, have been studied.

#### 3.1.2. Cellulose and Cellulose Derivatives

**Figure 5:**
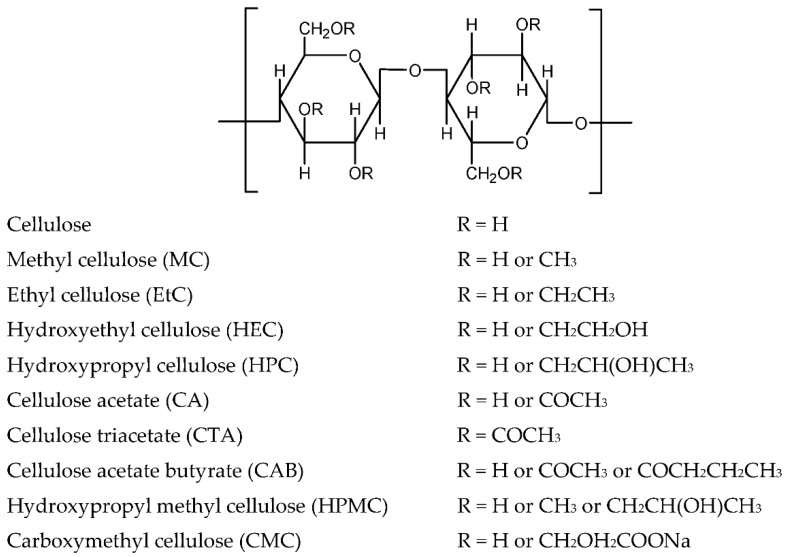
Representative units of cellulose and cellulose derivatives.

Cellulose is by far the most abundant and highly important renewable material on earth. It is the basic component of plant cell walls that has structural and skeletal functions. Cellulose has a high molecular weight and contains a linear homopolysaccharide polymer that consists of β-d-glucopyranose units in the ^4^C_1_ confirmation joined by (1→4) glycosidic linkage. The repeating element is made up by two anhydroglucose units. Cellulose exists in the form of microfibrils with a helical organization that contains crystalline and amorphous regions. The proportion of these regions varies depending on the microscopic level of the fiber assembly [15]. Cellulose derivatives can be formed by partially or totally reacting the three hydroxyl groups present in the anhydroglucose unit with various reagents. In fact, many types of cellulose derivatives have been studied, such as methyl cellulose, ethyl cellulose, hydroxyethyl cellulose, hydroxypropyl cellulose, cellulose acetate, cellulose triacetate, cellulose acetate butyrate, hydroxypropyl methyl cellulose, and carboxymethyl cellulose. Figure 5 shows the structures of general cellulose and cellulose derivatives.

Cellulose and cellulose derivatives have been widely applied in numerous applications. They are used as membranes for separation, as binders for drugs, a film coating agent, barrier films, textile applications, and many others [2]. The function of cellulose and cellulose derivatives as hosts in a polymer electrolyte system has been reported by many researchers. By far, cellulose and its derivatives, as presented in Figure 5, have been studied with respect to polymer electrolyte. The initial study was reported in 2001 for hydroxyethyl cellulose. Following that, research was actively conducted by incorporating various types of cellulose derivatives, along with the addition of salts, ionic liquids, plasticizers, and inorganic fillers.

#### 3.1.3. Chitosan

**Figure 6:**
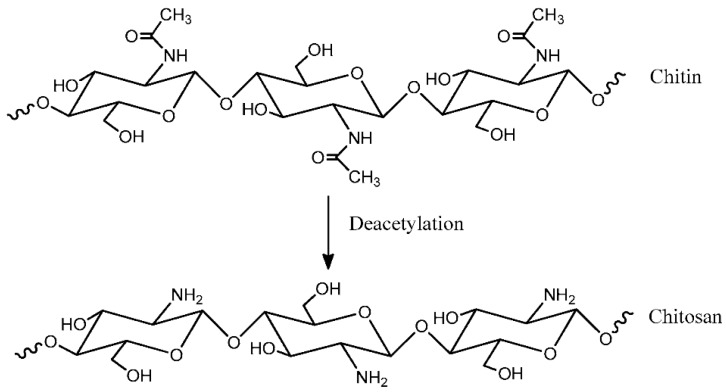
Molecular structure segment of chitin and chitosan.

Chitosan has received considerable attention in the polymer field. It shows many interesting properties, such as being non-toxic, biodegradable, and biocompatible. Chitosan consists of 1,4 linked-2-deoxy-2-aminoglucose, which is generated from the deacetylation reaction of chitin. Chitin refers to a natural polysaccharide that can be found in various fungi and the exoskeleton of arthropods [16] such as shrimps, crabs, and lobsters. Figure 6 shows the molecular structure segment of chitin and chitin deacetylation to generate chitosan [17].

The application of chitosan has been investigated in the medical field [18], water treatment studies [19], and in food packaging materials [20], to name a few. Interestingly, this polymer can also be applied in polymer electrolyte applications. Chitosan has several polar groups, such as hydroxyl and amino groups, that can act as donors and form complexes with inorganic salts [16]. Chitosan is an amorphous polymer and its glass transition temperature is reported to be ~200 °C [21]. Owing to these criteria, chitosan may serve as a polymer host for salt solvation. In fact, chitosan is the first biopolymer that has been studied for polymer electrolyte applications, reported in 1995. By far, chitosan is also the most widely studied biopolymer for this purpose. The literature portrays that the conductivity of native chitosan without salt is obtained at approximately 10^−9^ S/cm [22]. Other types of modified chitosan that have been studied are acetylated chitosan, chitosan acetate, oxipropylated chitosan, hexanoyl chitosan, carboxymethyl chitosan, N-phthaloyl chitosan, sulfonated chitosan, lauroyl chitosan, phosphorylated chitosan, and N-Succinyl chitosan. A wide variety of salts and acid dopants have been applied to improvise the ionic conductivity of the electrolyte system. The incorporation of ionic liquids began in 2010 and many more following that. Several studies have investigated the conductivity of chitosan doped with different types of salts, ionic liquids, plasticizers, and fillers.

#### 3.1.4. Gum

Gums are materials classified under polysaccharides with high commercial importance. They are present in many plant, animal, marine, and microbial sources [23]. Gums are available as raw powders. Table 2 lists the sources of common gums and their overall structure. The physiochemical properties of gums are determined by the chemical nature and the molecule shapes. All gums have one common similarity, which is the ability to thicken water and aqueous systems, though the rheological properties of the systems might vary depending on the types of gums being used [24]. Gums have gained many applications in various fields. For instance, in the food industry, gums are used as thickening, emulsifying, and gelling agents. In addition, they have also been applied as adhesives, binders, flocculants, and clarification aids [3]. In this section, gums that originate from marine algae and higher plants are discussed, while gums obtained from microorganisms are described in the latter part of this review.

##### Agar

**Figure 7:**
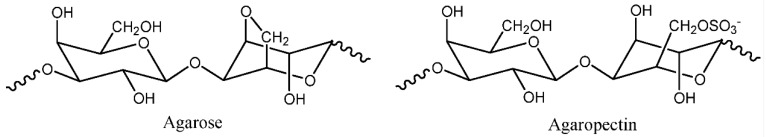
Representative structure of agarose and agaropectin.

Agar is a hydrocolloid material that is naturally obtained from the extraction of red seaweed, made up of a mixture of two polysaccharides, which are agarose and agaropectin. Agarose is a linear polymer and the predominant component of agar that makes up 70% of the mixture composed of the agarobiose repeating unit. Agarobiose refers to a disaccharide made up of D-galactose and 3,6-anhydro-L-galactopyranose [25]. Although both compounds share a similar galactose-based backbone, agaropectin contains acidic side groups, such as sulphate and pyruvate, whereas agarose has a neutral charge [26]. Figure 7 shows the representative structures of agarose and agaropectin [25]. The presence of numerous oxygen atoms in the agar structure satisfies the requirement of being a polymer host for an electrolyte, as it can be the possible site for complexation to take place.

The early use of agar in electrochemical studies was limited to the preparation of salt bridges in developing a reference electrode. In 2005, Kasem et al. looked into the suitability of agar as a polymer electrolyte host. The study investigated the electrochemical behavior of the electron redox system by using an agar/KCl gel electrolyte [27]. Since then, a few studies have examined agar-based electrolytes, which were prepared either in solid or gel form. Although agar is not as extensively studied as compared to cellulose or chitosan, various types of salts as an ion conductor and weak acids as a proton conductor have been investigated. The inclusion of ionic liquids, plasticizers, and nanoparticle fillers has been evaluated as well. So far, the highest room temperature ionic conductivity was achieved at 10^−3^ S/cm for an agar-based electrolyte, which is comparable to a liquid electrolyte. The performance of the agar-based electrolyte has been tested for DSSC, ECD, and fuel cell applications.

##### Carrageenan

**Figure 8:**
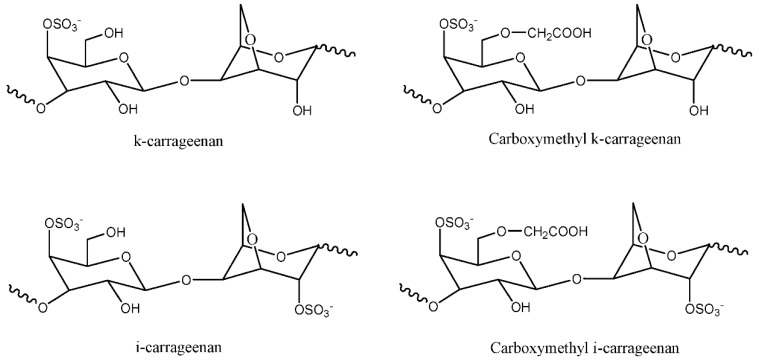
Representative units of κ-carrageenan, i-carrageenan, CMC κ-carrageenan, and CMC i-carrageenan.

Carrageenan is a linear sulphated polysaccharide polymer extracted from a type of marine red seaweed called *Rhodophyceae*. It can be classified into three main types: (a) Kappa (κ)-carrageenan that possesses one sulphate per disaccharide, (b) iota (ι)-carrageenan with two sulphates per disaccharide, and (c) lambda (λ)-carrageenan with three sulphates per disaccharide. It has galactose repeating units and 3,6-anhydrogalactose, both sulphated and non-sulphated, amalgamated by alternating α-(1,3) and β-(1,4) glycosidic links. Figure 8 portrays the representative units of carrageenan [28]. This polymer has been used extensively in the food, cosmetic, and pharmaceutical industries [22]. Studies pertaining to PE have, so far, looked into kappa and iota carrageenan, while none have researched lambda carrageenan.

The initial study concerning the potential of carrageenan in the polymer electrolyte field was reported by Mobarak et al. in 2012 [29]. The team prepared κ-carrageenan and a carboxymethyl (CMC) κ-carrageenan-based electrolyte via a solution casting method with a 1% (*v*/*v*) aqueous acetic acid solution. The room temperature ionic conductivity of 5.34 × 10^−7^ S/cm was achieved for κ-carrageenan. Interestingly, upon the modification to CMC κ-carrageenan, the conductivity increased three magnitudes to 2.02 × 10^−4^ S/cm. This enhancement was attributed to the fact that modification increased the amount of oxygen in the system, hence providing greater vacancies for protons or cations to coordinate. Another study was conducted by the same team utilizing CMC κ-carrageenan and ɩ-carrageenan with different ratios of lithium nitrate (LiNO_3_) salts (5–30 wt%). The highest ionic conductivity for CMC κ-carrageenan was obtained at 30 wt% LiNO_3_ salt with 5.85 × 10^−3^ S/cm, while the best conductivity of ɩ-carrageenan was recorded at 5.51 × 10^−3^ S/cm at 20 wt% of salt [22]. Another study concerning κ-carrageenan was conducted by Rudziah et al. In their study, carboxymethyl κ-carrageenan (CMKC) was blended with carboxymethyl cellulose (CMC). The cellulose was extracted from kenaf fiber and modified to produce CMC. The films of CMKC/CMC blend were prepared via the solution casting method with various ratios. They explained that the increase in conductivity was related with the increase in segmental motion and the fraction of the amorphous region [30]. The carrageenan-based electrolyte has been tested for various applications, such as DSSC, ECD, super-capacitors, and fuel cells.

##### Pectin

**Figure 9:**
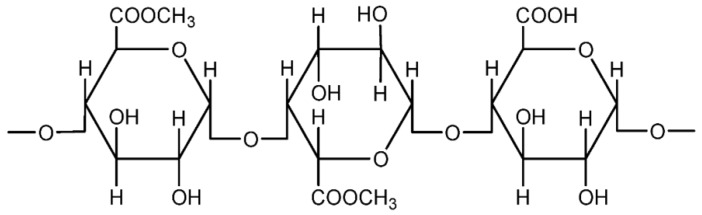
Representative unit of pectin.

Pectin is one type of polymer that is not widely explored in the polymer electrolyte field. It is a natural polymer, present naturally in the cell walls of terrestrial plants, and is abundant in vegetables and fruits. Citrus fruits, such as oranges and limes, contain substantial amounts of pectin. At present, the major sources are citrus peels and apple pomace, which are the by-products from the extraction of citrus and apple juices [31]. Typically, pectin is used in food products as it has beneficial effects upon the health of the consumer. Commercial pectin exists as a white to light brown powder. Pectin is usually applied as a gelling agent for food production. Chemically, pectin is a complex polysaccharide that is composed mainly of D-galacturonic acid resides in α-(1-4) chain (65 wt%). Pectin is also a group of substances which forms gel when dissolved in water under suitable conditions [32]. Figure 9 shows the representative unit of pectin [33].

The study of pectin-based polymer electrolytes was initiated in 2009 by Andrande et al. Pectin was doped with a fixed amount of LiClO_4_ and plasticized with glycerol for 0–70 wt%. The outcome reflected a good transparency feature with the pectin-based electrolyte film, which would be hardly achievable by a solid electrolyte. The best ionic conductivity value of 4.7 × 10^−4^ S/cm was obtained for the sample plasticized with 68 wt% of glycerol [33].

##### Guar gum and Gum Arabic

**Figure 10:**
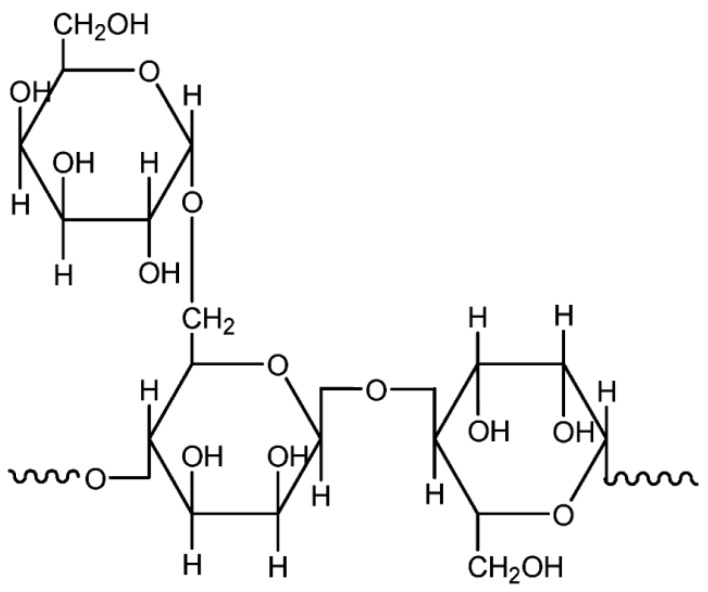
Representative unit of Guar gum.

Guar gum is processed from the endosperm of seeds from the cluster bean, *Cyamopsis tetragonolobus,* which belongs to the Leguminosae family. It contains a complex of polysaccharides called galactomannan, which is made up of D-galactose and D-mannose [34]. Figure 10 illustrates the representative unit of Guar gum [35]. This polymer contains an abundance of hydroxyl groups and tends to form hydrogen bonds when added to water. It is mainly used as an additive in food, pharmaceuticals, paper, textiles, and the cosmetics industry [36]. The application of guar gum as a polymer electrolyte began in 2014 initiated by Sudhakar et al. They prepared a solid polymer electrolyte of Guar gum/LiClO_4_/glycerol system and obtained a high room temperature ionic conductivity of 2.2 × 10^−3^ S/cm [37]. Meanwhile, the effects of the addition of ionic liquid and filler were studied by other researchers [35,38].

Gum Arabic, which is also known as Acacia gum, refers to the tree gum exudate of the Acacia tree. It is a highly branched polysaccharide and contains glycoprotein components. Gum Arabic is abundantly available and primarily used as an emulsifier, stabilizer, and thickening agent. The study of gum Arabic was performed by Khalid and Hartono for a supercapacitor application. They prepared a gel-like electrolyte by mixing gum Arabic with ortho-phosphoric acid. The gel electrolyte demonstrated excellent conductivity and supercapacitive performance.

#### 3.1.5. Gelatin

**Figure 11:**
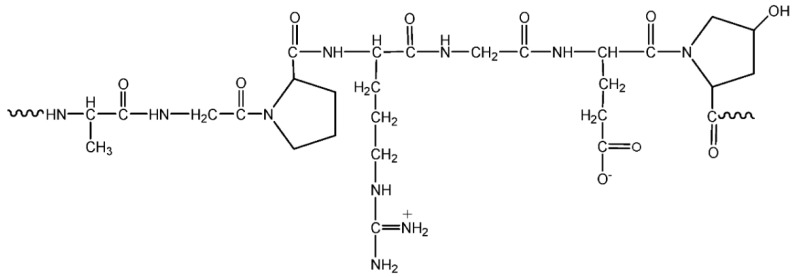
Representative unit of gelatin.

Gelatin is a soluble protein substance derived from collagen, a natural protein present in bonds, cartilage, and skin. The main source of gelatin is from bovine and porcine animals, but it also can be extracted from fish and poultry. Gelatin properties are influenced by several factors, such as the source, animal age, and collagen type. It has widespread applications, for example, emulsifiers, foaming agents, biodegradable packaging materials, and colloid stabilizers [39]. Gelatin is a polydisperse protein that is composed of a mixture of different chain types with varying molecular weights [40]. Figure 11 shows a representative unit of gelatin [41]. The study of gelatin-based polymer electrolytes started way back in 2007 by Diogo F et al [42]. The solid polymer electrolyte system was made up of gelatin/glycerol/acetic acid with the best room temperature ionic conductivity achieved at 10^−5^ S/cm. Subsequently, various types of salts have been investigated. The electrolyte system has also been tested for EDC and DSSC.

#### 3.1.6. Natural Rubber

**Figure 12:**
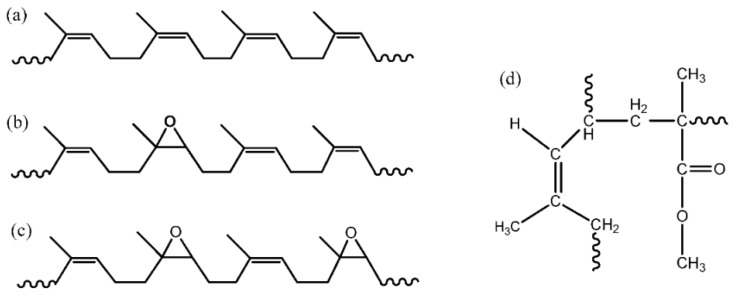
Structure of (**a**) natural rubber, (**b**) ENR-25, (**c**) ENR-50, and (**d**) MG-30 and MG-49.

Natural rubber is an elastic polymer obtained from the latex of rubber trees, made up of thousands of repeating isoprene units (C_5_H_8_)_n_. Natural rubber is produced from thousands of different plant species. In industrial applications, the most important source of natural rubber derives from the *Hevea brasiliensis* tree, which is mostly planted in the South-East Asia and Western Africa regions. Natural rubber has several distinctive properties, such as a low glass transition temperature, as well as good elasticity and adhesion characteristics. It has been widely applied in various applications, from household goods to the high end automotive industries [43]. Nonetheless, in a polymer electrolyte system, the natural rubber itself does not have any polar group in the structure to facilitate the ion mobility. Thus, modified natural rubber such as epoxidized natural rubber (ENR), ENR-25 and ENR-50 (where 25 and 50 represent the epoxy groups), as well as poly(methyl methacrylate)-grafted natural rubber (MG), MG-30 and MG-49 (where 30 and 49 indicate the percentages of methyl methacrylate grafted to the natural rubber), as shown in Figure 12, have been introduced to overcome the shortcoming.

The study of natural rubber-based polymer electrolytes was initiated by Yoshizawa et al. [44] in the year 2000. They blended natural rubber with polyethylene oxide via a solution casting method to produce a solid film electrolyte. The room temperature ionic conductivity obtained was 10^−6^ S/cm. Consequently, the focus shifted to natural rubber derivatives such as MG-30, MG-49, ENR-25, and ENR-50. Derivatives MG-30 and MG-49 were prepared by polymerizing a methyl methacrylate monomer in latex so that the polymer chains become attached to the rubber molecules. Meanwhile, ENR can be chemically modified from natural rubber, wherein some of the unsaturated group is converted into epoxide groups and randomly distributed [45]. Apart from being used as a single polymer host, natural rubber and its derivatives are blended with other polymers to enhance the properties of the electrolyte system.

### 3.2. Polymers Chemically Synthesized from Bio-Derived Monomers

The second type of bio-based polymer refers to polymers that are chemically synthesized from naturally-derived monomers. It is practically possible to produce tailor-made polymers with highly versatile properties using monomers. Nevertheless, studies pertaining to this type of polymer are only limited to poly(lactic acid) and some vegetable oil-based polyurethanes.

#### 3.2.1. Poly(lactic acid)

**Figure 13:**
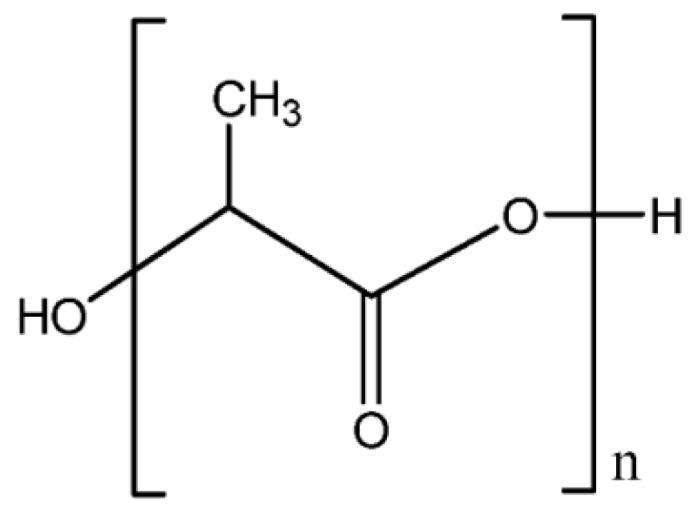
General structure of polylactic acid.

Poly(lactic acid) (PLA) is a linear aliphatic thermoplastic biodegradable polyester derived from two major pathways, namely, ring opening of lactide or direct polycondensation of lactic acid, a monomeric precursor obtained from renewable resources. The monomer is produced by a fermentation process of sugar feedstock, such as dextrose or chemical synthesis. Sugar feedstock can be obtained either directly from sources (sugar cane, sugar beet) or through conversion of starch from corn, potato, wheat, rice, or agricultural wastes. Figure 13 presents the general structure of poly (lactic acid).

Several studies have elaborately reported on PLA electrolytes. In a study, a PLA-based electrolyte was prepared via the solution casting method [46]. The outcomes showed that the conductivity of pure PLA at room temperature was 9.46 × 10^−12^ S/cm. Ethylene carbonate was added as the plasticizing agent. The incorporation of LiClO_4_ salt as the main ion carrier into the PLA/EC system enhanced the conductivity to 1.44 × 10^−6^ S/cm. The addition of SiO_2_ as a ceramic filler further enhanced the conductivity of the PLA electrolyte system to 1.29 × 10^−5^ S/cm. Subsequently, Chew [47] improvised a PLA-based electrolyte by incorporating aluminum oxide (Al_2_O_3_) as another type of ceramic filler. A similar composition was prepared as the previous PLA/EC/LiClO_4_ system and looked into various fillers. Based on the output, the inclusion of 4% Al_2_O_3_ displayed the highest conductivity at 2.07 × 10^−5^ S/cm. Osinska-Broniarz et al. [48] investigated the performance of PLA blended with poly 3-hydroxybutyrate (PHB) as a gel polymer electrolyte. The PLA/PHB blend polymer was prepared via the electrospinning method. The PLA/PHB membrane was then soaked in an electrolyte that consisted of lithium hexafluorophosphate (LiPF_6_) in a mixture of ethylene carbonate and dimethyl carbonate. A maximum room temperature ionic conductivity of 1.5 × 10^−5^ S/cm was obtained at a 70:30 weight ratio of PLA to PHB. In another study, a ternary polymer electrolyte based on PLA, an ionic liquid (Pyr_14_TFSI), and LiTFSI salt was prepared by Osada et al. [49] The materials were mixed by using the hot-pressed technique and melted together without including any solvents. The PLA/Pyr_14_TFSI/LiTFSI system achieved a conductivity of 2.1 × 10^−4^ S/cm at 60 °C.

#### 3.2.2. Vegetable Oil-Based Polyurethane

**Figure 14:**
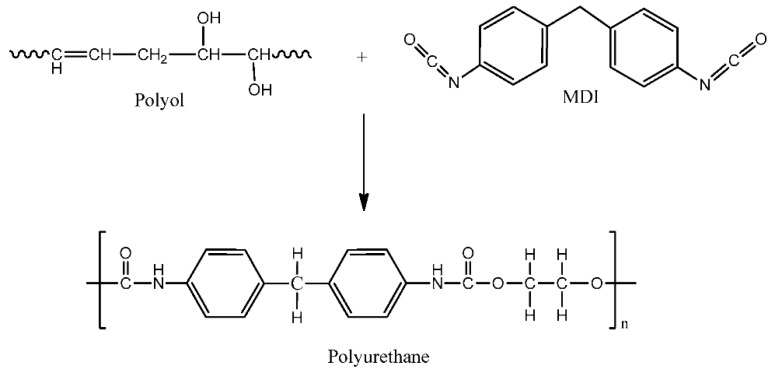
The reaction of polyol and MDI to produce polyurethane.

Vegetable oils are an excellent alternative to petrochemical feedstock. They can be used as a reliable starting material to produce new polymers. Vegetable oils are derived from plant sources, thus they are natural, abundant, and renewable. They can be classified into edible and non-edible oils. The most common vegetable oils include soybean oil, palm oil, sunflower oil, rapeseed oil, jatropha oil, and castor oil, to name a few. Vegetable oils are made up of long carbon chains and the main constituent is known as triglycerides. Some oils contain carbon-to-carbon double bonds (unsaturation site) that can be converted into the desired functional groups through chemical synthesis. For the polymer electrolyte purpose, palm oil, jatropha oil, and castor oil have been used as the raw material to prepare polyurethane (PU). Figure 14 shows the reaction of a vegetable oil-based polyol with the isocyanate group to produce polyurethane [50].

Su’ait et al. prepared palm oil-based polyurethane as a solid polymer electrolyte. In their study, palm kernel oil polyol (PKO-p) was reacted with 2,4′-methylene diphenyl diisocyanate (2,4′MDI) to produce PU. The electrolyte was prepared via the solution casting method with the inclusion of lithium iodide as the dopant salt at 10–30 wt% concentration, with ethylene carbonate as the plasticizing agent at a fixed amount of 20 wt%. The best room temperature ionic conductivity of 7.6 × 10^−4^ S/cm was obtained at 25 wt% LiI salt [51]. Another study of palm-based PU electrolyte was conducted by Daud et al., in which PKO-p was mixed with 2,4′-MDI in acetone at room temperature. The effect of various concentrations of LiCF_3_SO_3_ salt was evaluated. The highest room temperature conductivity was 1.6 × 10^−5^ S/cm obtained at 30 wt% LiCF_3_SO_3_ [8].

Castor oil-based polyurethane was synthesized by Salmiah et al. Castor oil is a great alternative to be used in generating PU as it is a non-edible oil. In their study, castor oil polyol was reacted with 4,4’-diphenylmethane diisocyanate (MDI) to produce PU. The electrolyte was prepared by mixing PU with lithium iodide (LiI) and sodium iodide (NaI) salt from 0–40 wt% salt concentration. The maximum ionic conductivity at room temperature was recorded at 30 wt% for both salts, with values 1.78 × 10^−6^ and 4.28 × 10^−7^ S/cm for LiI and NaI, respectively. They suggested that the PU/LiI system exhibited higher conductivity, when compared to PU/NaI due to the smaller cation size of Li^+^ than Na^+^. The sizes of Li^+^ and Na^+^ cations were 0.76 and 1.02 Å, respectively. Theoretically, a smaller cation size generates higher conductivity due to the higher mobility of the cation [52].

Jatropha oil is another example of a non-edible oil that has been used to prepare polyol for polyurethane production. Mustapa et al. prepared a solid polymer electrolyte from jatropha oil-based polyurethane doped with LiClO_4_ and ethylene carbonate. The highest conductivity of 1.29 × 10^−4^ S/cm was achieved at 25 wt% of LiClO_4_ salt.

### 3.3. Polymers Produced by Microorganisms

The direct production of bio-based polymers can be achieved by using microorganisms or genetically modified bacteria. In fact, a wide range of bio-based polymers with material properties suitable for industrial applications can and have been synthesized. This section will discuss some of the polymers that have been investigated as a host polymer electrolyte.

#### 3.3.1. Bacterial Cellulose

Bacterial cellulose is mainly used in the food industry, as well as in biomedical and cosmeceutical applications. It is produced by acetic acid bacteria in synthetic and non-synthetic media via oxidative fermentation [53]. Some cellulose-producing bacteria are *Acetobacter*, *Rhizobium*, *Agrobacterium*, and *Sarcina*. One important cellulose-producing bacterium is *Acetobacter xylinum,* which is the most efficient synthesizer of bacterial cellulose. The chemical structure of bacterial cellulose is similar to that of plant cellulose, except for the physical and chemical properties [54]. Bacteria cellulose possesses unique characteristics over plant cellulose, such as (1) absence of lignin and hemicellulose, (2) high degree of polymerization, and (3) extremely high water-holding capacity and excellent biodegradability. Despite this, the current price of this bacterial cellulose is still considered as too high for certain applications. The study of bacterial cellulose polymer electrolytes only started in 2015, and since then, only two studies have been reported [55,56]. Both studies successfully recorded high ionic conductivity similar to that of liquid electrolytes.

#### 3.3.2. Gellan Gum and Xanthan Gum

**Figure 15:**
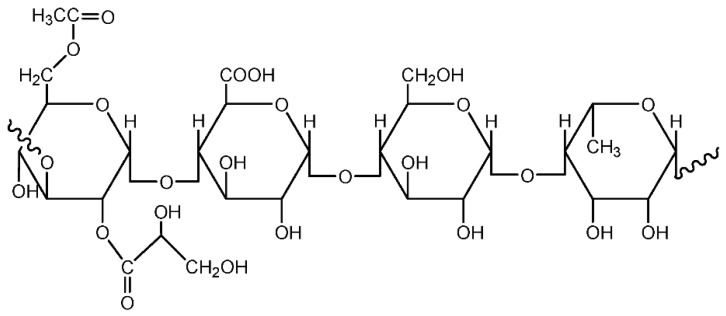
Representative unit of Gellan Gum.

Gellan gum is an extracellular, anionic polysaccharide made up of tetrasaccharides that consist of two glucoses, namely, one glucuronic acid and one rhamnose ring. It is produced from the fermentation of *Sphingomonas elodea* (ATCC 31461) by inoculating a fermentation medium with the microorganism. It is available in two forms, specifically high acyl (native gellan) and low acyl [57]. While the low acyl gellan is a firm, brittle, and non-elastic gel, the high acyl gellan on the other hand is a soft, elastic, and non-brittle gel. The gellan gum is mainly studied in the ophthalmology field for lenses with drug delivery properties [58]. One important property of gellan gum is the high thermal stability that can reach up to 120 °C and its thermal reversibility [59]. Figure 15 illustrates the representative units of gellan gum [58]. Gellan gum has been investigated as a polymer host doped with various types of salts and acid dopants. The initial study, undertaken in 2012, looked into applications for electrochemical devices.

**Figure 16:**
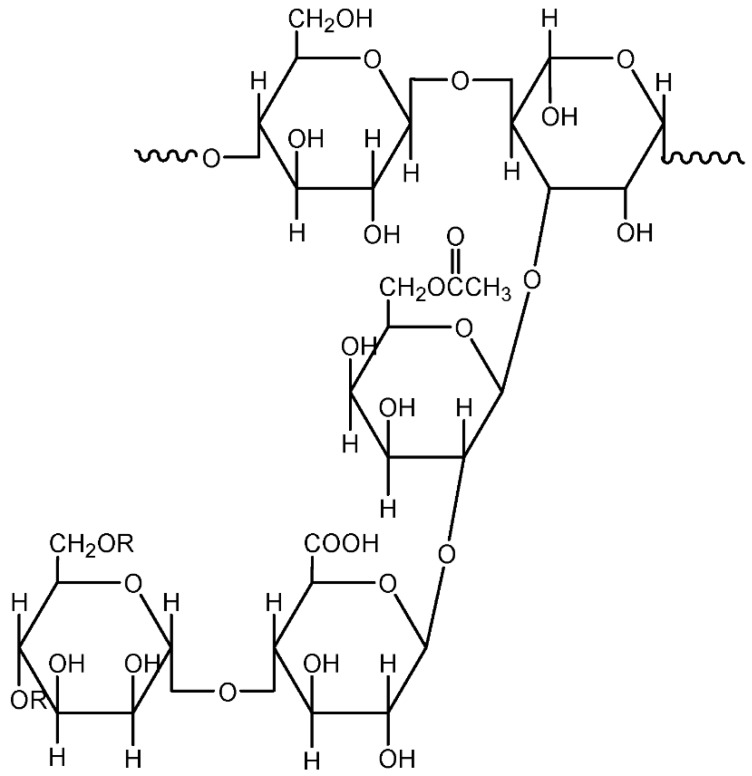
Representative unit of xanthan gum.

Xanthan gum is a high molecular weight polysaccharide produced via the microbiological fermentation of sugar cane/corn by the microorganism *Xanthomonas campestris*. Xanthan gum has excellent thermal stability, as well as good solubility and stability, thus it is suitable for use in the food, cosmetic, and pharmaceutical industries. Xanthan is an acidic polymer with the shape of a five-fold helix, made up of pentasaccharide subunits to form a cellulose backbone with trisaccharide side-chains composed of mannose (β-1,4) glucuronic acid, (β-1,2) mannose attached to alternate glucose residues in the backbone by α-1,3 linkages [23,60]. Figure 16 portrays the representative units of xanthan gum [61]. Only two studies have been reported regarding xanthan gum-based electrolytes. This particular electrolyte system displayed exceptional conductivity after being tested for dye sensitized solar cell and supercapacitor applications [23,61].

### 3.4. Development of Bio-Based Polymer Electrolyte

The use of bio-based polymers in the polymer electrolyte field is not a new concept. In fact, they have been used for a long time; however, only in the past three decades have they been extensively investigated in this field. Generally, each of the bio-based polymer hosts studied has its advantages and limitations. The advantages of these polymers are obvious, including the renewability, availability, and environmentally friendly nature. In spite of that, they have some shortcomings in terms of economic and technical aspects. The cost of said materials are relatively higher than the conventional petroleum-based polymers. Some technical issues like hydrophilic character and poor mechanical properties have hampered their applications. Thus, the properties must be improved in order for such electrolytes to be applied commercially. The main challenge is to produce an electrolyte system with high conductivity, whilst maintaining the electrochemical, thermal, and mechanical properties. Efforts have been taken to achieve this objective via several approaches, such as blending the polymer with other compatible polymers, the incorporation of fillers and the addition of plasticizers. Blending techniques aim to obtain new and unique materials with additional properties without sacrificing their original properties [62]. In general, polymer blend refers to mixture of at least two substances, polymers or copolymers, where the ingredient content is above 2 wt%. This method is applied because of its simple preparation and its ease of control over physical properties by compositional change [63].

Meanwhile, the addition of a plasticizer could improve the conductivity of a polymer electrolyte by reducing the glass transition temperature that facilitates the mobility of ions within the medium and dissociating ion aggregates [16]. Further, a high value of the dielectric constant of a plasticizer could solvate more salt, thereby increasing the number of free mobile ions [64]. The incorporation of organic/inorganic fillers could reduce the crystallinity and enhance both the mechanical and electrochemical properties. It has been discovered that the conductivity is strongly dependent on the particle size and concentration of the filler. Small-sized particles at low quantities are favorable and promote an enhanced conductivity [65]. In addition, room temperature ionic liquids (RTIL) have garnered interest as a substitute to organic solvents. RTIL is a molten salt containing bulky and asymmetric organic cations and contains highly delocalized charge inorganic anions. RTIL has some interesting features, such as excellent thermal and chemical stabilities, relatively high ionic conductivity, non-volatile, non-flammable, and wider electrochemical potential window [10].

The collective data of prior finding on bio-based polymer electrolytes using different types of bio-based polymer are shown in Table 3. The result was classified based on their source, origin, application, physical, and electrochemical properties. Various systems have been investigated by introducing different types of polymer hosts, salts, plasticizers, fillers, and ionic liquids. By far, starch, cellulose, and chitosan are the most widely studied and reported bio-based material in the polymer electrolyte field. The trend shows that the choice of salt used commonly depends on the intended end-use application. Different types of plasticizer have been used to enhance the ionic conductivity, such as glycerol, glucose, sorbitol, urea, formamide, glutaraldehyde, ethylene carbonate, propylene carbonate, and so on. Nano-oxide materials are a common choice to be used as fillers. Meanwhile, a wide variety of ionic liquids has been explored. From the findings, it is possible for the bio-based polymer electrolyte to achieve a room temperature ionic conductivity of 10^−2^ to 10^−3^ S cm^−1^, which is similar to the conductivity of the liquid electrolyte. Besides, gel electrolytes show better performance in comparison to the solid type. For a deep understanding, a complete test on electrochemical, thermal, and physical properties of the electrolyte is necessary to conduct in order to improve their performance in actual applications. Therefore, for the purpose of reliable electrochemical device applications, the bio-based polymer electrolytes should possess these characteristics: (1) Ionic conductivity (≥10^−4^ S cm^−1^); (2) high ionic transference number (t_ion_ ~1); (3) high chemical, thermal, and electrochemical stability; (4) good mechanical strength; and (5) good compatibility with the electrodes.

### 3.5. Application of Bio-Based Polymer Electrolytes

Bio-based polymer electrolytes have been tested in various electrochemical devices. This review focuses on the use of these electrolytes in batteries and DSSCs applications. Batteries have been widely used as energy supplies for portable devices, wearable electronics, and electric vehicles. Conventional batteries are made up of a cathode and anode, a separator to prevent physical contact between the two electrodes, and an electrolyte system. The most common anode materials used in batteries are titanium oxide, graphite, alloys, metal oxides, pure metal foils, etc. Meanwhile, the cathode materials used are vanadium oxide, molybdenum oxide, manganese oxide, silicates, LiCoO_2_, LiFePO_4_, LiMn_2_O_4_, etc. [376]. The electrolyte used in batteries’ construction is typically organic liquid electrolytes. The electrolyte is one of the key components that determine the battery’s performance which is related to the charging/discharging capacity, cycling performance, and current density [48]. In principle, when a battery is being charged, the ion moves from the cathode to the anode through the electrolyte, and during discharge, the ion will move back from the anode to the cathode. As discussed in the previous section, liquid electrolytes have a fundamental limitation for long-term operation due to their safety issues on evaporation and leakage, environmental concern, and restricted battery design. Hence, the best path is by replacing the conventional liquid electrolyte with all solid-state polymer electrolytes. Based on the literature, several studies on bio-based polymer electrolytes for lithium-ion batteries have been documented. The polymer systems include cellulose [146,155,160,161,165,169,177,181,182], chitosan [200,257], and natural rubber [360,367]. Despite the excellent performance, lithium-ion batteries rely on ultimately scarce and expensive resources. A sodium-ion battery is rather an interesting alternative as it is available abundantly at a very cheap cost. However, the use of bio-based polymer electrolytes in sodium-ion batteries is still in the early stage compared to lithium-ion batteries. A study on cellulose-based electrolyte on sodium-ion batteries was conducted by Colò and co-workers. The system shows good thermal stability and a wide electrochemical stability window [162]. Proton battery is another alternative to the lithium-ion battery that has been progressively studied. The electrochemical window for a proton battery is generally low, within the range of 1 to 2 V. Despite that, the availability of low-cost proton conductors has made proton batteries a good alternative. Some studies on bio-based polymer electrolytes for proton batteries have been reported on cellulose [129,133,179], chitosan [206,229,275], and natural rubber-based systems [350].

DSSC is the third generation of solar cells invented in 1991 by a team led by Gratzel [377]. Similarly to the previous generation of solar cells, DSSC converts sunlight energy directly into electrical energy through photovoltaic effects. DSSCs are interesting in regard to their remarkable advantages, such as low-cost production, robustness, colorful appearance, and possible flexibility. A typical DSSC consists of four components, which are a photoanode, a dye sensitizer, an electrolyte, and a counter electrode. The photoanode consists of a dye-coated nanocrystalline semiconductor oxide on a conducting substrate. It acts as a roadway for the electrons coursing through the cell. The counter electrode is usually a film of graphite or platinum. An electrolyte containing a redox couple fills the gap between the electrodes. The redox mediator is usually an organic solvent containing a redox system, such as an iodide/triiodide (I^⁻^/I^3⁻^) couple [378,379]. The use of a bio-based polymer electrolyte in place of the conventional organic solvent electrolyte could solve the leakage, corrosion, and stability issues often caused by the liquid-type electrolyte. Based on the literature, the I^−^/I^3−^ is the most efficient and widely used redox couple for bio-based polymer electrolyte in DSSC. Various iodide salts have been tested, such as LiI, NaI, KI, NH_4_I, etc. Some past study of bio-based polymer on DSSC is tabulated in Table 4. By far, the highest power conversion efficiency was achieved for cellulose-based electrolyte at 7.55% [139]. Despite this achievement, there are still some gaps that hinder the commercialization of bio-based polymer electrolytes in commercial application. Challenges that need to be addressed include the stability performance of the electrolyte overtime usage and the suitability of said materials in the selected application.

## 4. Summary and Outlook

Based on the literature available to date, a variety of bio-polymers have been explored by researchers, and the number of studies keeps on expanding, particularly over the last few decades. Similar to conventional petrochemical-based polymer electrolytes, bio-based polymers also suffer from low ionic conductivity when compared to liquid electrolytes. In fact, many studies have attempted to address this limitation. Researchers have suggested a number of ways to tackle this shortcoming by introducing fillers, plasticizers, and polymer blending methods. Nevertheless, the literature is still lacking in terms of the evaluation of the shelf life performance of bio-based electrolytes. This is another point of view that demands further investigation. As for applications, some of the bio-based polymer electrolytes have been tested in dye-sensitized solar cells, super-capacitors, and batteries. Despite the various types of bio-based materials that have been investigated as polymer electrolytes, they have yet to attain the status of commercial viability. Hence, extensive studies are still required to develop a system to achieve a level of performance that is comparable to the conventional liquid electrolytes. One interesting approach is to use computational and molecular modeling to understand the fundamental aspects of the materials. Such tools will provide important information, especially on the conduction mechanism, and can be used to assist and support the interpretation of experiments. Future work in this area will be very interesting as it will provide an in-depth understanding of the theoretical principle of the polymer electrolyte. From the preceding review, proper designs based on carefully selected materials and methods are expected to improve the bio-based polymer electrolytes performance.

## Figures and Tables

**Table 1:** Various types of bio-based polymers, their sources, and uses [3].

Bio-Based Polymer	Source	Uses
Starch	Sago, corn, tapioca, potato, rice	Adhesives, thickener and stabilizer in foods, and bio-plastics
Cellulose	Plants, bacterial	Paper, textile, and wood manufacturing
Chitin/chitosan	Shrimp, crab, lobster, shell fish	Cosmetics, foods, pharmaceutics
Oils	Palm oil, castor oil, soybean oil, canola oil	Resins, coatings, and adhesives
Pectin	Citrus fruits	Additives in food industry, pharmaceutics
Latex	Rubber tree, guayule shrubs	Medical, adhesives

**Table 2:** Sources of common gums and their overall structure.

Source		Gum	Structure
Marine algae		Agar, alginates, carrageenan	Linear, un-branched molecules
Higher plants	Extracts	Pectin	Linear, un-branched molecules
	Seeds	Guar gum	Linear molecules with short branches
	Exudates	Gum Arabic	Branch-on-branch molecules
Microorganism		Gellan, Xanthan	Linear molecules with short branches

**Table 3:** Summary of prior study on bio-based polymer electrolytes.

Polymer	Electrolyte System	State	Electrochemical Properties				Physical Properties		Device	Ref.
			σ (s/cm)	I-TN	Stability (V)	E_a_	T_g_ (°C)	Structural		
Starch	Corn starch–LiClO_4_–glycerol	Solid	7.9 × 10^−5^	–	–	–	−58	Amorph	–	[14]
	Corn starch–LiClO_4_–glycerol	Solid	5.0 × 10^−5^	–	–	–	–	–	–	[66]
	Corn starch–LiClO_4_–glycerol	Gel	10^−4^	–	–	0.35 eV	–	–	–	[67]
	Corn starch–NaCl–glucose	Solid	-	–	–	–	–	–	–	[68]
	Corn starch–NaCl–glycerol		-	–	–	–	–	–	–	
	Corn starch–NaCl–sorbitol		-	–	–	–	–	–	–	
	Corn starch–NaCl–urea		-	–	–	–	–	–	–	
	Corn starch–NaCl–formamide		10^−3^	–	–	–	–	–	–	
	Corn starch–LiClO_4_–glycerol	Solid	6.1 × 10^−5^	–	–	–	–	–	–	[69]
	Cassava starch–LiClO_4_–glycerol		8.4 × 10^−5^	–	–	–	–	–	–	
	Starch–NH_4_NO_3_	Solid	2.8 × 10^−5^	–	–	0.41 eV	–	–	–	[12]
	Corn starch–LiClO_4_–glycerol	Solid	1.1 × 10^−4^	–	–	–	−75	–	–	[70]
	Corn starch–LiTFSI–AmIm][Cl]	Solid	4.2 × 10^−4^	–	–	–	–	–	–	[71]
	Corn starch–LiPF_6_–[BmIm][PF_6_]	Solid	1.5 × 10^−4^	–	–	–	–	–	–	[72]
	Arrowroot starch–NaI–glutaraldehyde	Solid	6.7 × 10^−4^	0.95	–	–	–	–	–	[73]
	Tapioca starch/PEO–NH_4_NO_3_	Solid	2.8 × 10^−7^	–	–	–	–	Semi-cr	–	[63]
	Corn starch–LiPF_6_–[BmIm][Tf]	Solid	6.0 × 10^−4^	–	–	0.01 eV	−29	Amorph	–	[74]
	Sago starch–NH_4_Br	Solid	6.9 × 10^−9^	–	–	0.07 eV	-	–	–	[75]
	Corn starch–LiClO_4_–SiO_2_	Solid	1.2 × 10^−4^	–	–	–	87.1	–	–	[76]
	Corn starch–LiTFSI–[AmIm][Cl]	Gel	5.7 × 10^−2^	–	–	4.8 kJ/mol	–	–	–	[77]
	Corn starch–LiTFSI–DES	Solid	1.0 × 10^−3^	–	–	–	–	–	–	[78]
	Potato starch–NH_4_I	Solid	2.4 × 10^−4^	0.95	–	–	–	–	–	[13]
	Starch/chitosan–LiClO_4_–glycerol	Solid	3.7 × 10^−4^	–	–	0.52 eV	–	–	–	[79]
	Corn starch–AgNO_3_	Solid	-	–	–	0.71 eV	–	Amorph	–	[80]
	Corn starch–LiI–glycerol	Solid	9.6 × 10^−4^	–	–	0.16 eV	–	Amorph	–	[81]
	Corn starch–AgNO_3_	Solid	1.0 × 10^−9^	–	–	–	–	–	–	[82]
	Rice starch–LiI	Solid	4.7 × 10^−5^	–	–	0.41 eV	–	–	–	[83]
	Poly(styrene sulphonic acid)/starch–LiClO_4_–glycerol	Solid	5.7 × 10^−3^	–	–	–	–	–	–	[84]
	Potato starch–NaI–glutaraldehyde–PEG	Solid	1.8 × 10^−4^	0.99	–	–	75	–	–	[85]
	Corn starch–LiOAc–glycerol	Solid	1.0 × 10^−3^	–	2.1	0.14 eV	–	Amorph	–	[86]
	Sago starch–KI–I_2_	Solid	3.4 × 10^−4^	–	–	–	–	–	–	[87]
	Rice starch–LiI–MPII–TiO_2_	Solid	3.6 × 10^−4^	–	–	0.22 eV	–	Amorph	DSSC	[88]
	Corn starch–LiPF_6_–[BmIm][PF_6_]	Solid	1.5 × 10^−4^	–	2.9	–	–	–	Super-capacitor	[89]
	Corn starch–LiPF_6_–[BmIm][Tf]		3.2 × 10^−4^	–	3.1	–	–	–		
	Corn starch–LiClO_4_	Solid	1.6 × 10^−6^	–	–	0.64 eV	64	–	–	[90]
	Corn starch/chitosan–NH_4_I–glycerol	Solid	1.3 × 10^−3^	0.99	1.9	0.18 eV	–	–	–	[91]
	Starch/chitosan–NH_4_I	Solid	3.0 × 10^−4^	–	–	0.20 eV	–	Amorph	–	[92]
	Starch/chitosan–NH_4_Cl–glycerol	Solid	5.1 × 10^−4^	–	–	0.19 eV	−0.37	–	–	[93]
	Starch/chitosan–NH_4_Br–EC	Solid	1.4 × 10^−3^	0.92	1.8	0.17 eV	–	Amorph	EDLC	[94]
	Corn starch–LiClO_4_–SiO_2_	Solid	1.2 × 10^−4^	–	3.0	0.25 eV	–	–	EDLC	[95]
	Rice starch–LiI	Solid	4.7 × 10^−5^	–	–	–	−12	Amorph	DSSC	[96]
	Rice starch–NH_4_I		1.4 × 10^−4^	–	–	–	−38	Amorph		
	Rice starch–NaI		4.8 × 10^−4^	–	–	–	−42	Amorph		
	Rice starch–NaI–MPII	Solid	1.2 × 10^−3^	–	–	–	−58	Amorph	DSSC	[97]
	Corn starch–NH_4_Br–glycerol	Solid	1.8 × 10^−3^	0.98	1.6	0.11 eV	–	Amorph	–	[98]
	Corn starch–LiPF_6_–[BmIm][PF_6_]	Solid	2.0 × 10^−4^	–	2.9	–	–	Amorph	–	[99]
	Potato starch/PVA–KCl–glycerol	Solid	5.4 × 10^−5^	0.97	–	0.12 eV	–	Amorph	–	[100]
	Potato starch/chitosan–LiCF_3_SO_3_	Solid	7.1 × 10^−7^	–	–	–	–	–	–	[101]
	Potato starch/chitosan–LiCF_3_SO_3_–glycerol	Solid	1.3 × 10^−3^	–	–	0.11 eV	–	Amorph	–	[102]
	Corn starch–LiClO_4_–BaTiO_3_	Solid	1.8 × 10^−4^	–	3.1	–	17.2	–	EDLC	[103]
	Corn starch–LiTFSI	Solid	3.4 × 10^−4^	–	–	–	–	–	Battery	[104]
	Potato starch–Mg(C_2_H_3_O_2_)_2_–[BmIm][Cl]–glycerol	Solid	1.1 × 10^−5^	0.92	–	–	–	–	–	[105]
	Potato starch/Poly(vinyl alcohol)–LiBr–glycerol	Solid	10^−3^	–	–	–	–	–	–	[106]
	Potato starch/methylcellulose–LiClO_4_–glycerol	Solid	4.3 × 10^−4^	–	–	–	–	Amorph	–	[107]
	Starch/PVA-NH_4_SCN	Solid	1.3 × 10^−4^	–	–	–	–	Amorph	–	[108]
	Tapioca starch/chitosan–NH_4_NO_3_–[EmIm][NO_3_]	Solid	7.4 × 10^−5^	–	–	–	–	–	–	[109]
	Starch–NaCl	Gel	6.2 × 10^−2^	–	–	–	–	–	–	[110]
	Corn starch–LiClO_4_–glycerol	Solid	9.0 × 10^−3^	–	–	–	–	Amorph	–	[111]
	Potato starch–LiCF_3_SO_3_–[BmIm][Cl]–GO	Solid	4.8 × 10^−4^	–	–	–	–	Amorph	–	[112]
	Corn starch–NaClO_4_–glutaraldehyde	Solid	10^−2^	–	2.4	–	–	-	Super-capacitor	[113]
	Potato starch–NADES	Solid	2.9 × 10^−3^	–	–	–	–	Amorph	–	[114]
	Potato starch/methyl cellulose–NH_4_NO_3_–glycerol	Solid	1.3 × 10^−3^	0.98	1.8	–	−27.5	Amorph	EDLC	[115,116]
Cellulose	HEC/DPEO–LiClO_4_	Solid	2.1 × 10^−5^	–	–	0.17 eV	–	–	–	[117,118]
	HEC–LiClO_4_–glycerol	Solid	9.5 × 10^−5^	–	–	–	−60	Amorph	–	[14]
	Cellulose/PEO–LiCF_3_SO_3_	Solid	10^−7^	–	–	53 kJ/mol	–	–	–	[119]
	HPC/PEO–LiCF_3_SO_3_–PC	Gel	10^−3^	–	–	16 kJ/mol	–	–	–	[120]
	EO-EPI/nano-cellulose–LiClO_4_	Solid	1.6 × 10^−4^	–	–	–	–	–	–	[121]
	POE/nano-cellulose–LiTFSI–TEGDME	Solid	10^−6^	–	–	–	–	–	–	[122]
	HPC/Jeffamine–LiClO_4_	Solid	1.3 × 10^−5^	–	–	–	–	–	–	[123]
	POE/nano-cellulose–LiTFSI	Solid	10^−7^	–	–	–	–	–	–	[124,125]
	Cellulose acetate–LiClO_4_	Solid	4.9 × 10^−3^	–	–	–	–	–	Super-capacitor	[126]
	Ethyl cellulose–LiClO_4_-PC	Gel	6.5 × 10^−3^	–	–	0.18 eV	–	–	–	[127]
	PVDF–HPF/cellulose–LiPF_6_–EC/DMC	Solid	4.4 × 10^−3^	–	4.8	–	–	Semi-cr	–	[128]
	Cellulose acetate–NH_4_BF_4_–SiO_2_	Gel	7.9 × 10^−3^	–	–	–	–	–	Battery	[129]
	Cellulose triacetate–LiTFSI–Pyr_1,3_TFSI	Gel	10^−4^	–	–	–	–	–	–	[130]
	Cellulose–acrylic acid–[BmIm][I]	Gel	7.3 × 10^−3^	–	–	–	–	–	DSSC	[131]
	Cellulose acetate–NH_4_I–PC	Solid	1.2 × 10^−4^	–	–	–	–	–	–	[132]
	Cellulose acetate–NH_4_BF_4_–TiO_2_	Gel	1.4 × 10^−2^	–	–	–	–	–	Battery	[133]
	Cellulose acetate–NH_4_BF_4_–PEG	Solid	1.4 × 10^−5^	–	–	–	–	–	–	[134,135,136]
	CMC–DTAB	Solid	7.7 × 10^−4^	0.92	–	0.09 eV	–	Amorph	–	[137]
	PEG/network cellulose–LiClO_4_	Gel	10^−4^	–	4.7	–	–	Amorph	–	[138]
	CN-HPC–LiI–I_2_–MHII	Gel	2.5 × 10^−3^	–	–	–	–	Amorph	DSSC	[139]
	Cellulose acetate–LiTFSI–DES	Gel	2.6 × 10^−3^	–	–	4.23 kJ/mol	–	Amorph	–	[140,141]
	Cellulose acetate–LiTFSI–[AmIm][Cl]	Solid	1.8 × 10^−3^	–	–	–	–	Amorph	–	[142]
	Methyl cellulose–LiCF_3_SO_3_	Solid	2.1 × 10^−5^	–	–	–	–	Amorph	–	[143]
	Methyl cellulose–PEG–NH_4_NO_3_	Solid	10^−6^	–	2.4	–	–	Amorph	EDLC	[144]
	Methyl cellulose–KOH–DMC	Solid	10^−5^	–	–	–	–	–	–	[145]
	PE/PVDF/Cellulose acetate butyrate–LiPF_6_–EC/EMC	Gel	2.5 × 10^−3^	–	–	–	–	–	Battery	[146]
	PEO/CMC–NaI–I_2_–MPII	Gel	–	–	–	–	–	–	DSSC	[147]
	Cellulose acetate–LiBOB–GBL	Gel	5.4 × 10^−3^	–	4.7	–	–	–	–	[148]
	PEO/Network cellulose–LiClO_4_	Solid	8.0 × 10^−7^	–	5.0	–	–	Semi-cr	–	[149]
	PVDF–HFP/HPMC–LiPF_6_	Gel	3.8 × 10^−4^	–	5.0	–	–	Amorph	–	[150]
	Cellulose acetate–LiTFSI–[Amim][Cl]	Solid	4.7 × 10^−2^	–	–	1.25 kJ/mol	–	–	-	[151]
	CMC–LiClO_4_–PC	Gel	–	–	–	–	–	–	ECD	[152]
	MC–LiBOB	Solid	–	–	–	–	–	–	–	[153]
	MFC/BEMA/PEGMA–NaI–I_2_	Gel	–	–	–	–	–	Amorph	DSSC	[154]
	PVDF/Methyl cellulose–LiPF_6_–EC/EMC	Gel	2.0 × 10^−4^	–	–	–	–	–	Battery	[155]
	CMC–Citric acid	Solid	4.4 × 10^−7^	0.89	–	–	–	–	–	[156]
	MG-49/CMC–LiCF_3_SO_3_	Solid	3.3 × 10^−7^	–	–	–	–	Amorph	–	[157]
	Methyl cellulose–NaI	Solid	2.7 × 10^−5^	–	–	–	–	–	–	[158,159]
	MC–NWF–LiPF_6_–EC/DMC/EMC	Gel	2.9 × 10^−4^	–	–	–	–	–	Battery	[160]
	Cellulose acetate/PVDF–HFP–LiTFSI–TEGDME	Gel	5.5 × 10^−4^	–	4.7	–	–	–	Battery	[161]
	PEO/CMC–NaClO_4_	Solid	–	–	–	–	–	–	Battery	[162]
	Cellulose acetate–LiTFSI	Solid	5.6 × 10^−4^	–	–	–	–	Amorph	–	[163]
	HEC–H_3_PO_4_	Solid	4.1 × 10^−3^	–	–	0.12 eV	–	–	Super-capacitor	[164]
	CMC–LiPF_6_–EC/DMC/DEC	Gel	4.8 × 10^−4^	–	–	25.5 kJ/mol	–	–	Battery	[165]
	HPC–Bu_4_NBF_4_–PEG	Gel	3.5 × 10^−5^	–	–	–	−37	–	ECD	[166]
	Cellulose acetate–NH_4_I	Solid	10^−4^	–	–	–	–	–	DSSC	[167]
	CMC–CH_3_COONH_4_–BMATFSI	Solid	2.2 × 10^−3^	–	–	0.06 eV	–	–	-	[168]
	HEC–LiPF_6_–EC/DMC/DEC	Gel	1.8 × 10^−4^	–	–	3.57 kJ/mol	–	–	Battery	[169]
	CMC–NH_4_Cl	Solid	1.4 × 10^−3^	–	–	–	–	–	–	[170]
	CMC–(NH_4_)_2_CO_3_	Solid	7.7 × 10^−6^	–	–	–	–	Amorph	–	[171]
	CMC–NH_4_F	Solid	–	–	–	–	–	Semi-cr	–	[172]
	HPMC–Mg(CF_3_SO_3_)_2_–[BmIm][Tf]	Solid	2.4 × 10^−4^	–	–	1.28 eV	27.5	Amorph	–	[173]
	CMC–Oleic acid–glycerol	Solid	1.6 × 10^−4^	–	–	–	–	–	–	[174]
	Cellulose acetate–LiClO_4_–PC	Gel	5.3 × 10^−3^	–	–	–	–	–	ECD	[175]
	Cellulose acetate–NH_4_I–EC	Solid	10^−3^	–	–	–	–	Amorph	–	[176]
	PVDF/cellulose acetate butyrate/PE–LiPF_6_–EC/DMC/EMC–SiO_2_	Gel	2.9 × 10^−3^	–	5.2	–	–	–	Battery	[177]
	Cellulose acetate–LiNO_3_	Solid	1.9 × 10^−3^	–	4.1	0.16 eV	–	Amorph	ECD	[178]
	Cellulose acetate–NH_4_SCN	Solid	3.3 × 10^−3^	0.99	–	0.15 eV	113.7	Amorph	Battery	[179]
	Cellulose acetate–LiTFSI-BDG	Gel	2.9 × 10^−3^	–	3.8	–	–	Amorph	–	[180]
	Lignocellulose/potato starch–LiPF_6_–EC/DMC/EMC	Gel	1.3 × 10^−3^	–	–	12.7 kJ/mol	–	–	Battery	[181]
	Lignocellulose–PEG	Gel	3.2 × 10^−3^	–	–	–	–	–	Battery	[182]
	PVA/chitosan/CNC–Acetic acid	Solid	6.4 × 10^−4^	–	–	–	–	Amorph	Fuel cell	[183]
	HEC–Li_2_B_4_O_7_–glycerol	Solid	4.6 × 10^−3^	–	–	–	–	Amorph	–	[184]
	Cellulose acetate–NH_4_NO_3_	Solid	1.0 × 10^−3^	0.97	4.3	0.05 eV	111.6	Amorph	ECD	[185]
	CMC–(NH_4_)_2_CO_3_	Solid	7.7 × 10^−6^	0.98	–	0.21 eV	-	-	–	[186]
Chitosan	Acetylated chitosan–LiNO_3_	Solid	10^−4^	–	–	–	–	Amorph	Battery	[187]
	Chitosan acetate–NaI	Solid	4.9 × 10^−5^	–	–	–	–	–	Battery	[188]
	Chitosan–NaClO_4_	Solid	4.6 × 10^−2^	–	–	–	–	–	Battery	[189]
	Oxipropylated chitosan/polyether–LiTFSI	Solid	–	–	–	–	–	–	–	[190]
	Chitosan acetate–LiCF_3_SO_3_–EC	Solid	10^−5^	–	–	–	–	–	–	[21,191]
	Chitosan–KCl	Solid	–	–	–	–	–	–	–	[192]
	Chitosan acetate–LiCF_3_SO_3_–EC	Solid	1.3 × 10^−5^	–	–	–	–	–	Battery	[193]
	Chitosan acetate–LiOAc–palmitic acid	Solid	5.5 × 10^−6^	–	–	–	–	–	–	[194]
	Chitosan–LiCF_3_SO_3_–EC	Solid	5.5 × 10^−6^	–	–	0.44 eV	–	Amorph	–	[195]
	Chitosan–LiOAc–oleic acid	Solid	10^−5^	–	–	–	–	Amorph	–	[16]
	Chitosan–LiOAc–EC	Solid	7.6 × 10^−6^	–	–	–	–	–	–	[196]
	Chitosan acetate–LiN(CF_3_SO_2_)_2_–oleic acid	Solid	3.4 × 10^−6^	–	–	–	–	Amorph	–	[197]
	Chitosan–KOH	Solid	10^−2^	–	–	–	–	–	Fuel cell	[198]
	Chitosan acetate–NH_4_NO_3_	Solid	2.5 × 10^−5^	–	–	0.45 eV	–	Amorph	–	[17]
	Chitosan acetate–NH_4_NO_3_–Al_2_SiO_3_	Solid	2.1 × 10^−5^	–	–	–	–	–	–	[199]
	Hexanoyl chitosan–LiCF_3_SO_3_–EC/PC	Solid	1.1 × 10^−4^	–	–	–	–	–	Battery	[200,201]
	Chitosan acetate–NH_4_CF_3_SO_3_–DMC	Solid	10^−6^	–	–	0.60 eV	–	–	–	[202]
	Chitosan–LiOAc–oleic acid	Solid	1.1 × 10^−5^	–	–	0.29 eV	–	–	–	[203]
	Chitosan–LiOAc–palmitic acid		5.5 × 10^−6^	–	–	0.45 eV	–	–		
	Chitosan acetate–LiCF_3_SO_3_	Solid	–	–	–	0.38 eV	–	–	Super-capacitor	[204]
	Chitosan acetate–H_3_PO_4_			–	–	0.49 eV	–	–		
	Chitosan/glutaraldehyde–KOH	Solid	10^−2^	–	–	–	–	–	Fuel cell	[205]
	Chitosan acetate–NH_4_NO_3_–EC	Solid	9.9 × 10^−3^	–	–	–	–	–	Battery	[206]
	Hexanoyl chitosan–LiCF_3_SO_3_–EC	Gel	2.8 × 10^−5^	–	–	–	–	–	–	[207]
	Chitosan/PEO–LiTFSI	Solid	1.4 × 10^−6^	–	–	0.64 eV	–	–	–	[208]
	Chitosan acetate–NH_4_NO_3_–o-H_3_PO_4_	Solid	–	–	–	–	–	–	–	[209]
	Chitosan/PEO–NH_4_I–I_2_	Solid	4.3 × 10^−6^	–	–	–	–	–	DSSC	[210]
	Chitosan/PEO/pAPS–LiClO_4_	Solid	1.7 × 10^−5^	–	–	–	–	–	–	[211]
	Chitosan–HCl–glycerol	Solid	2.2 × 10^−5^	–	–	16.6 kJ/mol	−87	Amorph	–	[212]
	Chitosan–PVPA	Solid	–	–	–	0.32 eV	–	Amorph	–	[213]
	Chitosan acetate–NH_4_NO_3_–EC	Solid	10^−5^	–	1.8	0.10 eV	–	–	–	[214]
	Chitosan acetate–apidic acid	Solid	1.4 × 10^−9^	–	–	0.52 eV	–	–	–	[215]
	Chitosan acetate–NH_4_NO_3_–H_3_PO_4_–Al_2_SiO_3_	Solid	1.8 × 10^−4^	–	–	–	–	Amorph	Fuel cell	[216]
	Chitosan acetate/PEO–NH_4_NO_3_–ES	Solid	10^−4^	–	–	0.02 eV	–	–	–	[217]
	Chitosan acetate–CH_3_COONH_4_	Solid	2.9 × 10^−4^	–	–	0.19 eV	–	Amorph	–	[218]
	Hexanoyl chitosan–LiCF_3_SO_3_–EC–Al_2_O_3_	Solid	1.0 × 10^−4^	–	–	–	–	–	–	[219]
	Hexanoyl chitosan–LiClO_4_–TiO_2_	Solid	3.1 × 10^−4^	–	–	–	–	Amorph	–	[220,221]
	Hexanoyl chitosan–LiCF_3_SO_3_–DEC/EC	Solid	4.3 × 10^−5^	–	–	0.11 eV	–	Semi-cr	–	[222]
	Chitosan acetate–NH_4_I–EC	Solid	7.6 × 10^−6^	–	–	0.21 eV	–	–	–	[18]
	Chitosan–LiClO_4_–EC/PC	Gel	5.5 × 10^−3^	–	–	–	–	–	Super-capacitor	[223]
	Chitosan acetate–NH_4_I–[BmIm][I]	Solid	3.4 × 10^−5^	–	–	–	–	–	DSSC	[224,225]
	Chitosan acetate–NH_4_Cl	Solid	5.4 × 10^−3^	–	–	0.1 eV	–	Semi-cr	–	[226]
	Chitosan acetate–AgCF_3_SO_3_	Solid	–	–	–	1.16 eV	–	–	–	[227]
	Chitosan acetate–NaI-I_2_–[EmIm][SCN]	Solid	2.6 × 10^−4^	–	–	–	–	Amorph	DSSC	[228]
	PVA/chitosan acetate–NH_4_NO_3_	Solid	1.6 × 10^−3^	–	–	0.14 eV	–	Amorph	Battery	[229]
	Chitosan-g-PMMA–LiCF_3_SO_3_	Solid	4.1 × 10^−5^	–	–	–	110	–	–	[230]
	Chitosan/PEO–NH_4_I–I_2_–[BmIm][I]	gel	5.5 × 10^−4^	–	–	–	–	–	DSSC	[231]
	Chitosan acetate–NH_4_SCN–Al_2_O_3_	Solid	5.9 × 10^−4^	–	–	–	190	Semi-cr	–	[232]
	Hexanoyl chitosan–LiClO_4_–TiO_2_	Solid	–	–	–	–	–	–	–	[233]
	Chitosan acetate/PEO–NH_4_NO_3_	Solid	1.0 × 10^−4^	–	–	–	–	Semi-cr	–	[234]
	PVA/Chitosan–NH_4_NO_3_–EC	Solid	1.6 × 10^−3^	–	1.7	–	–	–	EDLC	[235]
	Chitosan–LiCF_3_SO_3_–EC/PC–SiO_2_	Solid	4.4 × 10^−5^	–	–	0.26 eV	–	–	–	[236]
	Chitosan/PVA–NH_4_I	Solid	1.8 × 10^−6^	–	–	0.38 eV	–	Amorph	–	[237]
	Chitosan acetate–glycerol	Solid	1.1 × 10^−5^	–	–	–	−70	–	–	[238]
	Phthaloyl chitosan–NH_4_SCN	Solid	2.4 × 10^−5^	–	2.1	0.08 eV	–	Amorph	–	[239]
	Chitosan–[CBIm][Cl]–I_2_	Solid	9.1 × 10^−3^	–	–	–	–	–	–	[240]
	Hexanoyl chitosan–LiClO_4_	Solid	4.2 × 10^−7^	–	–	–	–	–	–	[241]
	Hexanoyl chitosan–LiCF_3_SO_3_		4.1 × 10^−6^							
	Chitosan/PEO–NH_4_NO_3_	Solid	–	–	–	0.29 eV	–	–	–	[242,243]
	Nano-chitosan/PEO–LiCF_3_SO_3_	Solid	10^−3^	–	–	–	–	Semi-cr	–	[244]
	PEO/Chitosan–NH_4_I–I_2_	Solid	1.2 × 10^−5^	–	–	–	–	–	DSSC	[245]
	CMCh–ClCH_2_COOH	Solid	2 × 10^−7^	–	–	–	–	–	–	[246,247]
	Chitosan/PEO–LiClO_4_–EC/PC	Solid	1.1 × 10^−4^	–	–	0.12 eV	–	–	Super-capacitor	[248]
	Hexanoyl chitosan–LiCF_3_SO_3_–EC–Al_2_O_3_	Solid	–	–	–	–	–	–	–	[249]
	Chitosan–NH_4_Br–glycerol	Solid	2.2 × 10^−4^	–	–	0.20 eV	–	Amorph	–	[250]
	Chitosan–NH_4_SCN–Al_2_TiO_5_	Solid	2.1 × 10^−4^	–	–	–	–	Amorph	–	[251]
	Chitosan/PEO–NH_4_NO_3_–EC	Solid	2.1 × 10^−3^	–	1.75	0.18 eV	–	Amorph	EDLC	[64]
	Methyl cellulose/chitosan–NH_4_CF_3_SO_3_	Solid	5.0 × 10^−6^	–	–	–	–	–	–	[252]
	CMCh–NH_4_CF_3_SO_3_	Solid	8.9 × 10^−6^	–	0.8	–	–	–	–	[253]
	Chitosan–[EmIm][C_1_SO_3_]–glycerol	Solid	7.8 × 10^−4^	–	–	12.1 kJ/mol	–	–	–	[254]
	Chitosan–[EmIm][C_2_SO_3_]–glycerol		4.2 × 10^−4^	–	–	14.3 kJ/mol	–	–		
	Chitosan–[EmIm][C_4_SO_3_]–glycerol		1.5 × 10^−4^	–	–	16.7 kJ/mol	–	–		
	Hexanoyl chitosan–LiClO_4_–TiO_2_	Solid	3.1 × 10^−4^	–	–	0.08 eV	–	–	–	[255]
	Hexanoyl chitosan–LiClO_4_–SiO_2_		2.0 × 10^−4^	–	–	0.12 eV	–	–		
	Chitosan-g-PMMA–LiCF_3_SO_3_–EC	Solid	2.2 × 10^−4^	–	–	–	–	–	–	[256]
	Chitosan–LiTFSI–succinonitrile	Solid	0.4 ×10^−3^	–	4.7	–	–	Amorph	Battery	[257]
	CMC/chitosan–NH_4_Br	Solid	1.2 × 10^−5^	–	–	–	–	–	–	[258]
	Hexanoyl chitosan/polystyrene–LiCF_3_SO_3_–TiO_2_	Solid	2.8 × 10^−4^	–	–	–	–	Amorph	–	[259,260,261]
	PVA/chitosan–NH_4_Br	Solid	7.7 × 10^−4^	–	1.6	0.15 eV	–	Amorph	–	[262]
	Corn starch/chitosan–NH_4_I	Solid	3.0 × 10^−4^	–	–	0.20 eV	–	Amorph	–	[92]
	Chitosan/gold–LiClO_4_	Solid	7.2 × 10^−7^	–	–	–	–	Amorph	–	[263]
	Phosphorylated chitosan–LiClO_4_	Solid	1.4 × 10^−3^	–	–	–	–	–	–	[264]
	Chitosan–Oxalic acid	Solid	5.0 × 10^−7^	–	–	0.61 eV	–	Amorph	–	[265,266]
	N-Succinyl chitosan–LiClO_4_	Solid	8.0 × 10^−3^	–	–	–	–	–	–	[267,268]
	Hexanoyl chitosan–LiClO_4_–DMC	Solid	10^−4^	–	–	0.06 eV	–	–	–	[269]
	Hexanoyl chitosan–LiClO_4_–DMC–TiO_2_	Solid	4.1 × 10^−4^	–	–	–	–	Amorph	–	[270]
	Lauroyl chitosan/PMMA–LiCF_3_SO_3_–EC	Solid	7.6 × 10^−4^	–	–	–	–	Amorph	–	[271]
	NSB–Chitosan–NMPS–GO	Solid	8.9 × 10^−2^	–	–	4.57 kJ/mol	–	–	–	[272]
	Methyl cellulose/chitosan–NH_4_CF_3_SO_3_–[BmIm][TFSI]	Solid	4.0 × 10^−4^	–	–	–	–	–	–	[273]
	Starch/chitosan–NH_4_Cl–glycerol	Solid	5.1 × 10^−4^	0.97	1.65	–	–	Amorph	Battery	[274]
	Chitosan acetate–LiCl	Gel	2.9 × 10^−3^	–	–	0.20 eV	–	–	–	[275]
	Chitosan–[BmIm][OAc]	Solid	2.4 × 10^−3^	0.75	3.4	0.29 eV	35	Amorph	–	[276]
	Hexanoyl chitosan–LiClO_4_/TiO_2_	Solid	3.0 × 10^−4^	–	–	–	–	–	–	[277]
	PVA/chitosan–[BmIm][Br]	Solid	4.2 × 10^−2^	0.65	–	–	–	–	–	[278]
	PVA/chitosan–[EmIm][Cl]		5.5 × 10^−2^	0.70	–	–	–	–		
	Chitosan–NaCF_3_SO_3_–Al_2_O_3_	Solid	–	–	–	–	–	Amorph	–	[279]
	CMCh–DTAB	Solid	1.9 × 10^−6^	–	–	–	–	–	–	[280]
	Chitosan/PEO–NH_4_I	Solid	3.7 × 10^−6^	0.85	–	–	–	Amorph	DSSC	[281]
	Chitosan–LiClO_4_–ZrO_2_	Solid	3.6 × 10^−4^	0.55	–	–	–	Amorph	–	[282]
	Chitosan–perchloric acid	Solid	5.9 × 10^−4^	–	–	–	–	–	–	[283]
	N-phthaloyl chitosan–TPAI–I_2_–EC	Solid	5.5 × 10^−3^	–	–	0.11 eV	–	Amorph	DSSC	[284]
	Chitosan–oxalic acid	Solid	4.1 × 10^−5^	–	–	–	–	–	–	[285]
	Sulfonated chitosan–sulfonated GO	Solid	7.2 × 10^−3^	–	–	–	–	–	–	[286]
	Chitosan–LiCF_3_SO_3_–Al_2_O_3_	Solid	10^−6^	–	–	–	–	Amorph	–	[287]
	Chitosan–Ce(CF_3_SO_3_)_3_–glycerol	Solid	1.7 × 10^−5^	–	–	–	–	Amorph	–	[288]
	Chitosan–Eu(CF_3_SO_3_)_3_–glycerol	Solid	1.5 × 10^−6^	–	–	–	–	Amorph	–	[289]
	Chitosan–NaCF_3_SO_3_	Solid	2.4 × 10^−4^	–	–	0.3 eV	–	Amorph	–	[290]
	Chitosan/pectin–HCl	Solid	2.4 × 10^−3^	–	–	–	–	–	–	[291]
	Chitosan–Mg(CF_3_SO_3_)_2_–[EmIm][CF_3_SO_3_]	Solid	3.6 × 10^−5^	0.98	4.15	0.72 eV	–	–	–	[292]
	Hexanoyl chitosan–NaI	Solid	1.3 × 10^−6^	–	–	–	−24	Amorph	–	[293]
	Lauroyl chitosan–NaI		1.1 × 10^−8^	–	–	–	−10	Amorph		
	Chitosan–AgCF_3_SO_3_–Al_2_O_3_	Solid	–	–	–	–	–	–	–	[294]
	Chitosan–Tm(CF_3_SO_3_)_3_–glycerol	Solid	10^−5^	–	–	–	–	Amorph	ECD	[295]
	Chitosan–[EmIm][Eu(SCN)_4_]	Solid	1.3 × 10^−5^	–	–	–	–	Semi-cr	–	[296]
	Chitosan–[EmIm][SCN]	Solid	1.6 × 10^−3^	–	4.0	–	–	Amorph	–	[297,298]
Agar	Agar–Acetic acid	Solid	1.1 × 10^−4^	–	–	33.5 kJ/mol	–	Amorph	–	[11]
	Agar–LiI–I_2_–TiO_2_	Gel	5.1 × 10^−4^	–	–	–	–	–	DSSC	[299]
	Agar–LiI–I_2_–TiO_2_	Gel	4.0 × 10^−4^	–	–	–	–	–	DSSC	[300]
	Agar–Eu(pic)_3_–glycerol	Solid	1.6 × 10^−5^	–	–	–	–	Amorph	ECD	[301]
	Agar–LiClO_4_–glycerol	Gel	6.5 × 10^−5^	–	–	–	–	Amorph	ECD	[26]
	Agar–[EmIm][C_2_SO_4_]–glycerol	Solid	–	–	–	–	–	Amorph	ECD	[302]
	Agar–[EmIm][OAc]–glycerol		2.4 × 10^−5^	–	–	24.3 kJ/mol	–	Amorph		
	Agar–[Ch][OAc]–glycerol		–	–	–	–	–	Amorph		
	Agar–LiI–I_2_–NiO	Gel	–	–	–	–	–	–	DSSC	[303]
	Agar–LiI–I_2_–Fe_3_O_4_–PEG	Gel	2.9 × 10^−3^	–	–	–	–	–	DSSC	[304]
	Agar–LiI–I_2_–Fe_3_O_4_–SDS	Gel	–	–	–	–	–	–	DSSC	[305]
	Agar–LiI–I_2_–Fe_3_O_4_–PVP		–	–	–	–	–	–		
	Agar–LiI–I_2_–Fe_3_O_4_–TW-80		3.0 × 10^−3^	–	–	–	–	–		
	Bacto agar–NaI–I_2_	Gel	1.2 × 10^−3^	–	2.0	–	–	Amorph	–	[306]
	Agar–Mg(CF_3_SO_3_)_2_–glycerol	Solid	1.0 × 10^−6^	–	–	–	–	Amorph	ECD	[307]
	Agar–LiI–I_2_–TiO_2_	Gel	2.7 × 10^−3^	–	–	–	–	–	DSSC	[308]
	Agar–LiI–I_2_–Co_3_O_4_		4.4 × 10^−3^	–	–	–	–	–		
	Agar–LiI–I_2_–NiO		3.3 × 10^−3^	–	–	–	–	–		
	Agar–NiO–glycerol–acetic acid	Solid	5.2 × 10^−5^	–	–	–	–	Amorph	–	[65]
	Agar–LiClO_4_–glycerol	Solid	6.5 × 10^−8^	–	–	0.1 eV	–	Amorph	–	[25]
	Agar–KClO_4_–glycerol		9.1 × 10^−8^	–	–	0.1 eV-	–	Amorph		
	Agar–Acetic acid–glycerol		3.5 × 10^−8^	–	–	0.1 eV	–	Amorph		
	Agar–Lactic acid–glycerol		2.2 × 10^−8^	–	–	0.1 eV	–	Amorph		
	Agar–NH_4_NO_3_	Solid	6.6 × 10^−4^	0.99	–	0.12 eV	–	Amorph	Fuel cell	[309]
	Agar–Na_2_S/S–glycerol	Gel	1.8 × 10^−3^	–	–	–	–	–	DSSC	[310]
	Agar–NH_4_SCN	Solid	1.0 × 10^−3^	0.97	–	0.25 eV	55	Amorph	–	[311]
	Agar–NH_4_I	Solid	1.1 × 10^−4^	–	–	0.43 eV	–	Amorph	–	[312]
	Agar–KI–MPII	Gel	1.5 × 10^−3^	–	–	–	–	Amorph	DSSC	[313]
Carrageenan	Carboxymethyl κ-carrageenan–acetic acid	Solid	2.0 × 10^−4^	–	–	–	–	–	–	[29]
	κ-carrageenan–[Bmim]Cl	Solid	2.4 × 10^−3^	–	–	–	–	Amorph	–	[314]
	Carboxymethyl ɩ-carrageenan–acetic acid	Solid	4.9 × 10^−6^	–	–	–	–	Amorph	–	[315]
	Carboxymethyl κ-carrageenan/CMC–NH_4_I	Solid	2.4 × 10^−3^	0.99	2.0	0.01 eV	–	–	DSSC	[316]
	κ-carrageenan–TBAI–I_2_–TiO_2_	Solid	-	–	-	–	–	–	DSSC	[317]
	κ-carrageenan–TBAI–I_2_–Fe_2_O_3_		-	–	-	–	–	–		
	κ-carrageenan–TBAI–I_2_–halloysite clay		-	–	-	–	–	–		
	Carboxymethyl κ-carrageenan/CMC–acetic acid	Solid	3.3 × 10^−4^	–	2.75	–	−13.5	–	–	[30]
	Carboxymethyl κ-carrageenan–LiNO_3_	Solid	5.9 × 10^−3^	–	3.1	0.18 eV	–	–	–	[22]
	Carboxymethyl ɩ-carrageenan–LiNO_3_		5.5 × 10^−3^	–	3.0	0.38 eV	–	–		
	Carboxymethyl κ-carrageenan/CMC–LiI–I_2_	Solid	3.9 × 10^−3^	–	-	0.01 eV	−43.0	–	DSSC	[318]
	ɩ-carrageenan–NH_4_Br	Solid	1.1 × 10^−3^	–	2.1	0.18 eV	-	Amorph	Fuel cell	[319]
	k-carrageenan/PEDOT–PANI	Gel	–	–	–	–	–	–	Super-capacitor	[320]
	ɩ-carrageenan–NH_4_NO_3_	Solid	1.5 × 10^−3^	0.95	2.46	0.14 eV	64	–	ECD	[321]
Pectin	Pectin–LiClO_4_	Solid	4.7 × 10^−4^	–	–	–	–	Amorph	–	[33]
	Pectin–HCl–glutaraldehyde	Solid	2.5 × 10^−2^	–	–	–	–	Amorph	–	[322]
	Pectin–KCl–glycerol	Solid	1.5 × 10^−3^	–	–	–	–	Amorph	–	[323]
	Pectin–KCl–Ir(III)–glycerol		5.4 × 10^−5^	–	–	–	–	Amorph		
	Pectin–NH_4_Cl	Solid	4.5 × 10^−4^	–	–	–	–	Amorph	–	[324]
	Pectin–NH_4_Br		1.1 × 10^−3^	–	–	–	–	Amorph		
	Pectin–[N_1112(OH)_][NTf_2_]–glycerol	Solid	1.4 × 10^−6^	–	–	–	–	Amorph	–	[325]
Guar gum and gum arabic	Guar gum–LiClO_4_–glycerol	Solid	2.2 × 10^−3^	–	–	0.18 eV	–	–	–	[37]
	Guar gum–[BmIm][Cl]–PEDOT	Gel	10^−2^	–	–	–	–	–	–	[35]
	Guar gum–[BmIm][Cl]–P(AEMIBr)	Gel	10^−4^	–	–	–	–	–	–	[38]
	Gum Arabic–o-H_3_PO_4_	Gel	1.8 × 10^−2^	–	–	–	–	–	Super-capacitor	[326]
Gelatin	Gelatin–glycerol–formaldehyde–acetic acid	Solid	4.5 × 10^−5^	–	–	32.6 kJ/mol	–	–	–	[42]
	Gelatin–LiClO_4_–glycerol	Solid	10^−4^	–	–	0.35 eV	–	–	–	[327]
	Gelatin–LiBF_4_–glycerol	Gel	2.3 × 10^−5^	–	–	–	–	–	–	[41]
	Gelatin–LiClO_4_–glycerol	Gel	3.2 × 10^−5^	–	–	–	–	–		
	Gelatin–HCl–glycerol	Gel	5.4 × 10^−5^	–	–	–	–	–		
	Gelatin–Acetic acid–glycerol	Gel	8.7 × 10^−4^	–	–	–	–	–		
	Gelatin–LiClO_4_–EC/PC	Solid	2.0 × 10^−9^	–	–	–	–	–	–	[328]
	Gelatin–LiClO_4_	Solid	–	–	–	–	–	–	ECD	[329]
	Poly(acrylic acid-g-gelatin)/polypyrrole–KI–I_2_	Gel	1.4 × 10^−2^	–	–	10.3 kJ/mol	–	–	DSSC	[330]
	Gelatin–Acetic acid–glycerol	Solid	2 × 10^−5^	–	–	0.22 eV	–	–	–	[331]
	Gelatin–LiBF_4_–glycerol	Gel	1.5 × 10^−5^	–	–	43.1 kJ/mol	–	Amorph	–	[332]
	Gelatin–[Eu(pic)_3_]–glycerol	Solid	–	–	–	–	–	Amorph	ECD	[333]
	Gelatin–HCl–glycerol	Solid	4.0 × 10^−5^	–	–	23 kJ/mol	–	–	–	[334]
	Gelatin–[EmIm][OAc]	Solid	1.2 × 10^−4^	–	–	16.7 kJ/mol	–	Amorph	ECD	[335]
	Gelatin–LiI–I_2_	Solid	5 × 10^−5^	–	–	8 kJ/mol	−76	Amorph	ECD	[336]
	Gelatin–LiClO_4_–glycerol	Solid	10^−4^	–	–	–	–	–	–	[337,338]
	Gelatin–Glycerol	Solid	9.1 × 10^−3^	–	–	–	–	Amorph	–	[339]
	Gelatin–Zn(CF_3_SO_3_ )_2_	Solid	3.1 × 10^−10^	–	–	–	–	Amorph	–	[340]
	Gelatin–LiClO_4_–glycerol	Solid	1.1 × 10^−4^	–	–	9.37 kJ/mol	–	Amorph	–	[341]
	Gelatin–LiCl–glycerol		2.0 × 10^−4^	–	–	6.31 kJ/mol	–	Amorph		
	Gelatin–Mg(CF_3_SO_3_)_2_–glycerol	Solid	3.8 × 10^−10^	–	–	49.0 kJ/mol	–	Amorph	–	[39]
	Gelatin/Au–LiI–I_2_	Gel	2.2 × 10^−2^	–	–	–	77	–	DSSC	[342]
	Gelatin–[EmIm][N(CN)_2_]–glycerol	Solid	2.4 × 10^−3^	–	–	–	–	Amorph	ECD	[343]
	Gelatin–NaCl	Gel	8.5 × 10^−2^	–	–	–	–	–	–	[110]
Natural rubber	NR/PEO–LiBs-PEO_1000_	Solid	10^−6^	–	–	–	–	–	–	[44]
	ENR-25–LiCF_3_SO_3_–EC/PC	Solid	2.9 × 10^−4^	–	–	–	−43	–	–	[344]
	ENR-50–LiCF_3_SO_3_–EC/PC		1.3 × 10^−4^	–	–	–	−35	–		
	MG-49–LiCF_3_SO_3_–EC/PC		4.3 × 10^−4^	–	–	–	–	–		
	ENR-25/PEO–LiCF_3_SO_3_–EC/PC	Solid	10^−4^	–	–	–	–	–	–	[345]
	ENR-50/PEO–LiCF_3_SO_3_–EC/PC		10^−4^	–	–	–	–	–		
	MG-30–LiCF_3_SO_3_–EC–Al_2_SiO_5_	Solid	–	–	–	–	−41	–	–	[346]
	PVC/ENR-50–LiCF_3_SO_3_	Solid	3.6 × 10^−5^	–	–	–	–	–	–	[347]
	PMMA/ENR-50–LiCF_3_SO_3_		5.1 × 10^−5^	–	–	–	–	–		
	MG-30–LiCF_3_SO_3_	Gel	8.4 × 10^−4^	–	4.3	–	–	–	–	[348]
	MG-30–LiCF_3_SO_3_–EC		–	–	–	–	–	–		
	MG-30–LiCF_3_SO_3_–PC		–	–	–	–	–	–		
	PMMA/ENR-50–LiCF_3_SO_3_–EC	Solid	–	–	–	–	–	–	–	[349]
	MG-49–NH_4_CF_3_SO_3_–PC	Gel	3.3 × 10^−2^	–	–	–	–	–	Battery	[350]
	MG-49–LiBF_4_	Solid	2.3 × 10^−7^	–	–	–	–	Amorph	–	[351]
	MG-49–LiClO_4_		4.0 × 10^−8^	–	–	–	–	Amorph		
	MG-49–LiClO_4_–EC–TiO_2_	Solid	1.1 × 10^−3^	–	–	–	–	–	–	[352]
	MG-49–NH_4_CF_3_SO_3_–SiO_3_	Gel	7.6 × 10^−3^	–	–	–	–	–	–	[353]
	PMMA/MG-49–LiBF_4_	Solid	8.3 × 10^−6^	–	–	–	–	Amorph	–	[354]
	MG-49–NH_4_CF_3_SO_3_–PC	Gel	2.9 × 10^−2^	–	–	–	–	–	–	[355]
	MG-30–NH_4_CF_3_SO_3_–EC	Solid	10^−4^	–	–	–	–	Amorph	–	[356]
	PVC/LENR-50–LiClO_4_	Solid	2.3 × 10^−8^	–	–	–	–	Amorph	–	[357]
	ENR-50–Li_2_NH	Solid	3.5 × 10^−5^	–	–	–	–	–	–	[358]
	MG-49/Cellulose–LiCF_3_SO_3_	Solid	5.3 × 10^−7^	–	–	–	–	Amorph	–	[359]
	MG-30–LiCF_3_SO_3_–EC	Gel	9.0 × 10^−3^	–	4.2	0.14 eV	−77.2	–	Battery	[360]
	PVDF–HFP/MG-49–LiCF_3_SO_3_	Solid	2.0 × 10^−4^	–	3.0	0.14 eV	–	Semi-cr	–	[361,362,363]
	ENR-50–LiN(SO_2_CF_3_)_2_–EC/PC	Solid	2.6 × 10^−4^	–	–	–	−46.8	–	–	[364]
	PVDF/MG-49–NH_4_CF_3_SO_3_	Solid	6.3 × 10^−4^	–	4.0	–	–	Semi-cr	–	[365]
	MG-49/CMC–LiCF_3_SO_3_	Solid	3.3 × 10^−7^	–	2.7	–	–	–	–	[366]
	MG-49–LiCF_3_SO_3_–ZrO_2_/TiO_2_	Solid	1.2 × 10^−5^	–	–	0.10 eV	–	Amorph	Battery	[367]
	MG-30–LiCF_3_SO_3_	Solid	5.6 × 10^−3^	–	–	–	−43.9	–	–	[368]
	ENR-50–LiClO_4_	Solid	10^−5^	–	–	–	−22	–	–	[369]
	ENR-25/hexanoyl chitosan–LiN(CF_3_SO_2_)_2_–[EmIm][TFSI]	Solid	1.3 × 10^−6^	–	–	–	−31	–	–	[370]
	ENR-25–LiClO_4_	Solid	–	–	–	–	−42	–	–	[371]
Poly(lactic acid)	PLA–LiClO_4_–EC–Al_2_O_3_	Solid	2.1 × 10^−5^	–	–	–	–	Amorph	Battery	[47]
	PLA–LiClO_4_–EC–SiO_2_	Solid	1.3 × 10^−5^	–	–	–	–	Amorph	–	[46]
	PLA/PHB/PC–LiPF_6_–EC	Gel	1.5 × 10^−2^	–	4.4	–	69	Semi-cr	Battery	[48]
	PLGA/SY–TBABF_4_	Solid	10^−10^	–	–	–	–	–	LEC	[372]
	PLA–LiTFSI–Pyr_14_TFSI	Solid	–	–	–	–	0.1	Amorph	Battery	[49]
Vegetable oil-based polyurethane	Palm-based PU–LiCF_3_SO_3_	Solid	1.6 × 10^−5^	–	–	–	–	Amorph	–	[8]
	Palm-based PU–LiI–I_2_–EC	Solid	7.6 × 10^−4^	–	–	0.11 eV	–	Amorph	DSSC	[51]
	Castor oil-based PU–LiI	Solid	1.4 × 10^−6^	0.99	2.0	0.13 eV	−27.5	–	–	[52]
	Castor oil-based PU–NaI		4.3 × 10^−7^	0.98	1.8	0.22 eV	−26.1	–		
	Jatropha oil-based PU–LiClO_4_–EC	Solid	1.3 × 10^−4^	0.83	4.8	2.8 meV	–	Amorph	–	[50]
Bacterial cellulose	Bacterial cellulose–Nafion	Solid	5.6 × 10^−2^	–	–	–	–	–	Fuel cell	[55]
	Bacterial cellulose/PSSA–HCl	Solid	10^−3^	–	–	–	–	–	Fuel cell	[56]
Gellan and Xanthan gum	Gellan–LiCF_3_SO_3_	Solid	5.4 × 10^−4^	–	5.4	14.6 kJ/mol	–	Semi-cr	–	[58]
	Gellan–LiI–Glycerol	Solid	1.5 × 10^−3^	–	–	2.4 kJ/mol	–	–	–	[59]
	Gellan–[N_1112(OH)_][NTf_2_]–Er(CF_3_SO_3_)_3_	Solid	5.2 × 10^−6^	–	3.5	–	–	Semi-cr	ECD	[373]
	Gellan–KI–I_2_	Solid	2.5 × 10^−2^	–	–	0.24 eV	–	Amorph	DSSC	[374]
	Gellan–o-H_3_PO_4_	Gel	5.1 × 10^−3^	–	–	0.14 meV	–	–	EDLC	[375]
	Gellan–H_2_SO_4_		1.5 × 10^−3^	–	–	0.17 meV	–	–		
	Gellan–HCl		3.7 × 10^−4^	–	–	0.19 meV	–	–		
	Xanthan–PMII–I_2_–TBP–GSCN	Gel	–	–	–	–	–	–	DSSC	[61]
	Xanthan–LiClO_4_–glycerol	Gel	2.6 × 10^−3^	–	–	–	–	–	Super-capacitor	[23]
	Xanthan–Li_2_B_4_O_7_–glycerol		2.7 × 10^−2^	–	–	–	–	–		

(I-TN: Ionic transference number; Ea: Activation energy, Tg: Glass transition temperature; Amorph: Amorphous; Semi-cr: Semi-crystalline).

**Table 4:** The photovoltaic performance of DSSC observed in bio-based polymers–salt matrix.

Polymer	Electrolyte System	State	σ (s/cm)	J_SC_ (mA/cm^2^)	V_OC_ (V)	FF	η (%)	Ref.
Starch	Rice starch–LiI–MPII–TiO_2_	Solid	3.6 × 10^−4^	0.49	0.45	0.75	0.17	[88]
	Rice starch–LiI	Solid	4.7 × 10^−5^	–	–	–	–	[96]
	Rice starch–NH_4_I		1.4 × 10^−4^	–	–	–	–	
	Rice starch–NaI		4.5 × 10^−4^	2.40	0.48	0.67	0.78	
	Rice starch–NaI–MPII	Solid	1.2 × 10^−3^	4.78	0.57	0.76	2.09	[97]
Cellulose	Cellulose-g-acrylic acid–[BmIm][I]	Gel	7.3 × 10^−3^	12.65	0.71	0.611	5.51	[131]
	CN–HPC–LiI–I_2_–MHII	Gel	2.5 × 10^−3^	14.40	0.76	0.70	7.55	[139]
	PEO/CMC–NaI–I_2_–MPII	Gel	-	10.03	0.75	0.69	5.18	[147]
	MFC/BEMA/PEGMA–NaI–I_2_	Gel	-	15.20	0.76	0.61	7.03	[154]
Chitosan	Chitosan/PEO–NH_4_I	Solid	3.7 × 10^−6^	2.71	0.58	0.50	0.78	[281]
	N-phthaloyl chitosan–TPAI–I_2_–EC	Solid	5.5 × 10^−3^	12.72	0.60	0.66	5.00	[284]
Agar	Agar–LiI–I_2_–TiO_2_	Gel	-	10.96	0.54	0.57	4.74	[299]
	Agar–NH_4_I–I_2_–Glycerol	Gel	4.9 × 10^−3^	0.007	0.29	–	–	[380]
	Agar–LiI–I_2_–NiO	Gel	–	–	–	–	2.95	[303]
	Agar–MPII–PC–GuSCN–NMBI–I_2_	Gel	–	11.73	0.70	0.64	5.25	[381]
	Agar–AEII–PC–GuSCN–NMBI–I_2_			11.71	0.72	0.65	5.45	
	Agar–DAII–PC–GuSCN–NMBI–I_2_			11.53	0.70	0.62	4.97	
	Agar–APII–PC–GuSCN–NMBI–I_2_			11.84	0.70	0.60	4.96	
	Agar–LiI–I_2_–Fe_3_O_4_–SDS	Gel	–	3.18	0.66	0.62	1.29	[305]
	Agar–LiI–I_2_–Fe_3_O_4_–PVP		–	3.00	0.67	0.59	1.19	
	Agar–LiI–I_2_–Fe_3_O_4_–TW-80		3.0 × 10^−3^	5.00	0.70	0.53	1.83	
	Agar–KI–I_2_	Gel	9.0 × 10^−3^	3.27	0.67	0.24	0.54	[382]
	Agar–LiI–I_2_–TiO_2_	Gel	2.7 × 10^−3^	5.28	0.61	0.55	1.71	[308]
	Agar–LiI–I_2_–Co_3_O_4_		4.4 × 10^−3^	7.24	0.63	0.46	2.11	
	Agar–LiI–I_2_–NiO		3.3 × 10^−3^	6.20	0.62	0.52	2.02	
	Agar–Na_2_S/S–glycerol	Gel	–	10.75	0.58	0.47	2.97	[310]
	Agar–KI–MPII	Gel	1.5 × 10^−3^	9.28	0.46	0.50	2.16	[313]
Carrageenan	Carboxymethyl κ-carrageenan/CMC–NH_4_I	Solid	2.4 × 10^−3^	0.49	0.60	0.64	0.13	[316]
	κ-carrageenan–TBAI–I_2_–TiO_2_	Solid	–	1.83	0.75	0.55	0.76	[317]
	κ-carrageenan–TBAI–I_2_–Fe_2_O_3_		–	3.96	0.68	0.07	0.20	
	κ-carrageenan–TBAI–I_2_–halloysite clay		–	1.39	0.74	0.50	0.51	
	Carboxymethyl κ-carrageenan/CMC–LiI–I_2_	Solid	3.9 × 10^−3^	0.40	0.49	0.57	0.11	[318]
Gelatine	Poly(acrylic acid-g-gelatine)/polypyrrole–KI–I_2_	Gel	1.4 × 10^−2^	2.76	0.66	0.70	1.28	[330]
	Gelatine/Au–LiI–I_2_	Gel	2.2 × 10^−2^	4.94	0.65	0.60	1.97	[342]
Vegetable oil-based polyurethane	Palm-based PU–LiI–I_2_–EC	Solid	7.6 × 10^−4^	0.06	0.14	-	-	[51]
	Castor oil-based PU–NaI	Solid	4.3 × 10^−7^	3.60	0.49	0.46	0.80	[383]
	Palm-based PU–MPII	Gel	9.1 × 10^−4^	3.30	0.71	0.36	1.00	[384]
Gellan and Xanthan gum	Gellan–KI–I_2_	Solid	2.5 × 10^−2^	3.20	0.57	0.90	1.47	[374]
	Xanthan–PMII–I_2_–TBP–GSCN	Gel	–	–	–	–	4.78	[61]

(σ: Conductivity; J_SC_: Short-circuit current density; Voc: Open circuit potential; FF: Fill factor; η: Efficiency)

## Abbreviations

AEII	1-allyl-3-ethylimidadolium iodide
AgNO_3_	Silver nitrate
Al_2_O_3_	Aluminium oxide
Al_2_SiO_5_	Aluminium silicate
[AmIm][Cl]	1-allyl-3-methylimidazolium chloride
APII	1-allyl-3-propylimidazolium iodide
BaTiO_3_	Barium titanate
BDG	Diethylene glycol dibutylether
BEMA	Bisphenol A ethoxylate dimethacrylate
BMATFSI	Butyl-trimethyl ammonium bis(trifluoromethylsulfonyl)imide
[BmIm][Cl]	1-butyl-3-methylimidazolium chloride
[BmIm][I]	1-butyl-3-methylimidazolium iodide
[BmIm][OAc]	1-butyl-3-methylimidazoliumacetate
[BmIm][PF_6_]	1-butyl-3-methylimidazolium hexafluorophosphate
[BmIm][Tf]	1-butyl-3-methylimidazolium trifluoromethanesulfonate
[BmIm][TFSI]	1-butyl-3-methylimidazolium trifluoromethanesulfonyl imide
Bu_4_NBF_4_	Tetrabutylammonium tetrafluoroborat
Ce(CF_3_SO_3_)_3_	Cerium triflate
[Ch][OAc]	Trimethyl-ethanolammonium acetate
CH_3_COONH_4_	Ammonium acetate
CMC	Carboxymethyl cellulose
CMCh	Carboxymethyl chitosan
CNC	Cellulose nanocrystals
CN-HPC	Cyanoethylated hydroxypropyl cellulose
Co_3_O_4_	Cobalt oxide
DAII	1-3-diallylimidazolium iodide
DES	Deep eutectic solvent
DMC	Dimethyl carbonate
DPEO	Poly(ethylene oxice) diisocyanate
DSSC	Dye sensitized solar cell
DTAB	Dodecyltrimethyl ammonium bromide
EC	Ethylene carbonate
ECD	Electrochromic device
EDLC	Electrical double layer capasitor
EMC	Ethyl methyl carbonate
[EmIm][Br]	1-butyl-3-methylimidazolium bromide
[EmIm][Cl]	1-ethyl-3-methylimidazolium chloride
[EmIm][CF_3_SO_3_]	2-ethyl-3-methylimidazolium trifluoromethanesulfonate
[EmIm][C_1_SO_3_]	1-ethyl-3-methylimidazolium methylsulfonate
[EmIm][C_2_SO_3_]	1-ethyl-3-methylimidazolium ethylsulfonate
[EmIm][C_4_SO_3_]	1-ethyl-3-methylimidazolium butylsulfonate
[EmIm][C_2_SO_4_]	1-ethyl-3-methylimidazolium ethylsulfate
[EmIm][Eu(SCN)_4_]	1-ehtyl-3-methylimidazolium europium(III) tetrathiocyanate
[EmIm][OAc]	1-ethyl-3-methylimidazolium acetate
[EmIm][N(CN)_2_]	1-ethyl-3-methylimidazolium dicyanamide
[EmIm][NO_3_]	1-ethyl-3-methylimidazolium nitrate
[EmIm][SCN]	1-ethyl 3-methylimidazolium thiocyanate
[EmIm][TFSI]	1-ethyl-3-methylimidazolium bis(trifluoromethylsulfonyl)imide
ENR-25	25% epoxidized natural rubber
ENR-50	50% epoxidized natural rubber
EO-EPI	ethylene oxide-epichlorohydrin
Er(CF_3_SO_3_)_3_	Erbium triflate
ES	Ethylene sulphite
EtC	Ethyl carbonate
[Eu(pic)_3_]	Europium picrate
Eu(CF_3_SO_3_) _3_	Europium triflate
Fe_2_O_3_	Iron (III) oxide
Fe_3_O_4_	Iron (II,III) oxide
GBL	γ-butyrolactone
GO	Graphene oxide
GSCN	Guanidinium thiocyanate
H_2_SO_4_	Sulphuric acid
HCF	Hexacyanoferrate
HCl	Hydrochloric acid
HEC	Hydroxyethyl cellulose
HPC	Hydroxypropyl cellulose
HPMC	Hydroxypropyl methyl cellulose
I_2_	Iodine
KCl	Potassium chloride
KI	Potassium iodide
LENR-50	Liquid 50% epoxidized natural rubber
Li_2_B_4_O_7_	Lithium tetraborate
LiBF_4_	Lithium tetrafluoroborate
LiBoB	Lithium bis(oxalato) borate
LiBs	lithium benzenesulfonate
LiCF_3_SO_3_	Lithium trifluoromethanesulfonate
LiCl	Lithium chloride
LiClO_4_	Lithium perchlorate
LiI	Lithium iodide
LiN(CF_3_SO_2_)_2_	lithium trifluoromethane sulfonimide/lithium imide
LiNO_3_	Lithium nitrate
LiOAc	Lithium acetate
LiPF_6_	Lithium hexafluorophosphat
LiTFSI	Lithium bis(trifluoromethane sulfonyl)imide
MFC	Microfibrillated cellulose
MG-30	30% methyl methacrylate grafted natural rubber
MG-49	49% methyl methacrylate grafted natural rubber
Mg(C_2_H_3_O_2_)_2_	Magnesium acetate
Mg(CF_3_SO_3_)_2_	Magnesium triflat
MHII	1-methyl-3-hexylimidazolium iodide
MPII	1-methyl-3-propylimidazolium iodide
[N_1112(OH)_][NTf_2_]	*N*,*N*,*N*-trimethyl-*N*-(2-hydroxyethyl)ammonium bis(trifluoromethylsulfonyl)imide
NaCl	Sodium chloride
NaClO_4_	Sodium perchlorate
NADES	Ternary natural deep eutectic solvent (NADES)
NaI	Sodium iodide
Na_2_S/S	Poly sulphide solution
NH_4_BF_4_	Ammonium tetrafluoroborate
NH_4_Br	Ammonium bromide
NH_4_CF_3_SO_3_	Ammonium triflate
NH_4_Cl	Ammonium chloride
(NH_4_)_2_CO_3_	Ammonium carbonate
NH_4_I	Ammonium iodide
NH_4_NO_3_	Ammonium nitrate
NH_4_SCN	Ammonium thiocyanate
NiO	Nickel oxide
NMBI	N-methylbenzimidazole
NMPS	*N*,*N*-dimethylene phosphonic acid propylsilane
NR	Natural rubber
NSB-Chitosan	*N*-*o*-sulphonic acid benzyl chitosan
NWF	Nonwoven fabric
o-H_3_PO_4_	Ortho-phosphoric acid
PMMA	poly(methyl methacrylate)
PAN	Poly(acrylonitrile)
PANI	Polyaniline
pAPS	Poly(aminopropyltriethoxysilane)
PC	Propylene carbonate
PE	Polyethylene
PEDOT	Poly(3,4-ethylenedioxythiophene)
PEG	Poly(ethylene glycol)
PEGMA	Poly(ethylene glycol) methyl ether methacrylate
PEO	Poly(ethylene oxide)
PHB	Poly(3-hydroxybutyrate)
PLA	Poly(lactic acid)
PLGA	Poly(lactic-co-glycolic acid)
PMII	Propyl-methyl-imidazolium iodide
PMMA	Poly(methyl methacrylate)
P(AEMIBr)	Poly(1-[2-acryloylethyl]-3-methylimidazolium bromide)
POE	Poly(oxyethylene)
PSSA	Poly(4-styrene sulfonic acid) (PSSA)
PVA	Poly(vinyl alcohol)
PVC	Poly(vinyl chloride)
PVDF	Poly(vinylidene fluoride)
PVDF-HFP	Poly(vinylidene fluoride-hexafluoro propylene)
PVP	Poly(vinylpyrrolidone)
PVPA	poly(vinylphosphonic acid)
Pyr_14_TFSI	*N*-butyl-*N*-methylpyrrolidinium bis(trifluoromethane-sulfonyl) imide
SDS	Sodium dodecyl sulphate
SiO_2_	Silicon diioxide
SY	Super yellow
TBABF_4_	Tetrabutylammonium tetrafluorborate
TBAI	Tetrabutylammonium iodide
TBP	4-tert-butylpyridine
TEGDME	Tetra(ethylene) glycol dimethyl ether
TiO_2_	Titanium dioxide
Tm(CF_3_SO_3_)_3_	thulium triflate
TPAI	Tetrapropylammonium iodide
TW-80	Polysorbate 80
Zn(CF_3_SO_3_)_2_	Zinc triflate
